# Modelling Sorption and Transport of Gases in Polymeric Membranes across Different Scales: A Review

**DOI:** 10.3390/membranes12090857

**Published:** 2022-08-31

**Authors:** Eleonora Ricci, Matteo Minelli, Maria Grazia De Angelis

**Affiliations:** 1 Department of Civil, Chemical, Environmental and Materials Engineering (DICAM), Alma Mater Studiorum—University of Bologna, 40126 Bologna, Italy; 2 Institute for Materials and Processes, School of Engineering, University of Edinburgh, Edinburgh EH9 3FB, UK

**Keywords:** solubility, diffusivity, permeability, modelling, equations of state, transport models, molecular simulations, gas separation, polymers

## Abstract

Professor Giulio C. Sarti has provided outstanding contributions to the modelling of fluid sorption and transport in polymeric materials, with a special eye on industrial applications such as membrane separation, due to his Chemical Engineering background. He was the co-creator of innovative theories such as the Non-Equilibrium Theory for Glassy Polymers (NET-GP), a flexible tool to estimate the solubility of pure and mixed fluids in a wide range of polymers, and of the Standard Transport Model (STM) for estimating membrane permeability and selectivity. In this review, inspired by his rigorous and original approach to representing membrane fundamentals, we provide an overview of the most significant and up-to-date modeling tools available to estimate the main properties governing polymeric membranes in fluid separation, namely solubility and diffusivity. The paper is not meant to be comprehensive, but it focuses on those contributions that are most relevant or that show the potential to be relevant in the future. We do not restrict our view to the field of macroscopic modelling, which was the main playground of professor Sarti, but also devote our attention to Molecular and Multiscale Hierarchical Modeling. This work proposes a critical evaluation of the different approaches considered, along with their limitations and potentiality.

## 1. Introduction

A multitude of applications are associated with the sorption and transport of gases and vapors in polymeric materials, such as membrane separation, carbon capture, polymer production and processing, packaging, volatile organic compound detection, thin-film coating, and environmental protection [1,2,3,4,5,6,7,8].

Detailed knowledge of sorption and transport in realistic operating conditions is required in the majority of such applications. For instance, the design of membrane-based separation processes, which are a low-carbon, low-energy alternative to many conventional purification processes, requires the full understanding of gas and vapor solubility and transport behaviors with respect to, e.g., temperature and pressure, to correctly identify optimal conditions. Furthermore, assessment of membrane performance under mixed-gas conditions is of great importance, as multicomponent phenomena can greatly affect separation. An increasing number of process simulators can include and evaluate membrane units in process design, but the accuracy of material property predictions can be significantly improved [9].

One fundamental issue to take into account when dealing with polymeric membranes is that many high-performance materials for fluid separations, e.g., polyimides, are glassy, and their non-equilibrium nature makes the sorption and transport of fluids a function of their thermal, solvation, and mechanical history [10,11,12,13]. In such systems, sorption cannot be described based on equilibrium thermodynamics tools, e.g., activity coefficients or equation-of-state models.

Certain polymeric membranes are not amorphous but semicrystalline, while most models refer to disordered phases. The modeling of fluid sorption in semicrystalline materials is an interesting subtopic in this subject, but it is not treated here, as a comprehensive review of this topic appeared earlier this year [14].

Combining polymers with inorganic fillers in composite materials yields structures of undeniable interest in various applications due to their optimal mechanical and thermal properties. In the membrane separation field, they are conventionally named “mixed matrix membranes” and are formed by nano-sized particles dispersed in a polymer matrix. The particles can be dense and impermeable, affecting the membrane separation performance by modifying its internal morphology, or they can be porous structures that contribute to membrane separation ability with their intrinsic permeability and selectivity. For the first type of membranes incorporating impermeable fillers, the modeling of sorption and transport was reviewed some years ago when this type of structure was the most popular one [15]; for porous fillers or more generally mixed matrices, we refer the reader to more recent reviews [16,17,18].

In this work, we focus on modeling homogenous amorphous polymeric membranes. We start by presenting macroscopic models for gas solubility in rubbery polymers, namely, activity coefficient approaches, and Equations-of-State (EoS), and then follow with the Non-Equilibrium Theory for Glassy Polymers (NET-GP) that extends the prediction of sorption to glassy polymers. Other tools specifically developed for glassy polymers, but less generalized, are also presented, such as the Dual-Mode Sorption (DMS) and GAB equations, together with the more recent fractal model. Subsequently, molecular methods are described, and their strengths and limitations compared to macroscopic approaches are highlighted. We devote particular attention to the conditions needed for predictive calculations and extension to the multicomponent gas phase. For this reason, discussion of empirical correlations for sorption and transport of pure fluids in various families of polymers [19,20] falls outside the scope of this review.

## 2. Modelling Fluid Transport in Dense, Homogenous Polymeric Membranes: The Solution–Diffusion Model

Simulation of the separation properties of dense homogenous polymer membranes relies on the so-called solution–diffusion model, briefly recalled hereafter. The steady-state flux of a gas i across a membrane is experimentally observed to be proportional to the pressure gradient across the membrane [21]:

(1) $$N_{i}=P_{i}\frac{∆p_{i}}{l}$$

where $N_{i}$ is the transmembrane flux, which coincides with the diffusive flux $J_{i}$ in the case of a negligibly small concentration of the gas in the polymer, as often occurs in gas separation membrane applications, $∆p_{i}$ is the partial pressure difference of component i across the membrane, l is membrane thickness, and $P_{i}$ is the permeability coefficient. Therefore, the transport of small molecules in dense polymeric membranes is characterized by a permeability coefficient, which is defined as the pressure- and thickness-normalized flux of gas across the membrane. The permeability coefficient is thus introduced through an operative empirical definition. However, with a few assumptions, it can be shown that, as far as dense homogenous polymeric membranes are considered, its value can be correlated to more fundamental and predictable properties, namely the diffusivity and solubility of the fluid species in the polymeric material. Such development goes under the name of solution–diffusion model, which was formalized by Wijmans and Baker [21,22] and has emerged as the most widely accepted model for the description of transport in dialysis, reverse osmosis, gas permeation, and pervaporation.

The driving force for the diffusion of a penetrant i is the gradient of its chemical potential:

(2) $$J_{i}=-c_{i}L_{i}\frac{d\left(\mu_{i}/RT\right)}{dz}$$

where $J_{i}$ is the steady-state flux, $\frac{d\mu_{i}/RT}{dz}$ is the chemical potential gradient along z, $L_{i}$ is the penetrant mobility, also called self-diffusion coefficient, and $c_{i}$ is the penetrant molar concentration. The diffusive flux can be expressed using concentration as a driving force, which is the typical formulation of Fick’s law:

(3) $$J_{i}=-D_{i}\frac{dc_{i}}{dz}$$

In such a formulation, $D_{i}$ is the mutual binary diffusion coefficient of the fluid in the polymer, which is not only influenced by the penetrant mobility in the system, but also by thermodynamic effects.

This, in turn, can be correlated to the self-diffusivity $L_{i}$, for which calculation methods are more often available [23]:

(4) $$D_{i}≃{\left(\frac{\partial \ln f_{i}}{\partial \ln\omega_{i}}\right)}_{T,P}L_{i}$$

where $f_{i}$ represents gas fugacity, and $\omega_{i}$ is the mass concentration of the gas. Mutual diffusivity $D_{i}$ is obtained as the product of a kinetic factor, the mobility, or self-diffusivity, $L_{i}$, and a thermodynamic factor, i.e., the derivative of the fugacity with respect to concentrations, in brackets. The correction introduced by the thermodynamic factor to the diffusion coefficient is relevant in the case of nonlinear sorption isotherms, such as those typical of sorption of light gases in glassy polymers, whereas for polymers in the melt state, the effect is less marked.

It must be noted that diffusive flux coincides with total flux only at low concentrations of diffusing species in a non-swollen membrane [24]. When the membrane is highly swollen, a frame-of-reference correction [25,26], which accounts for convective flux in addition to the diffusive one given by Fick’s law, needs to be applied. Kamaruddin and Koros [27] showed that assuming negligible convective flux can lead to significant errors in multicomponent mixtures when the permeability of one component is much higher than that of others.

An alternative approach is to replace Fick’s law in the solution–diffusion model by the Maxwell–Stefan diffusive transport equation [28], which is based only on the relative velocities of the components of the system, bypassing the frame-of-reference problem. One drawback of this approach is that the concentrations of all permeants in the membrane material are required to calculate the permeant fluxes, which makes it of less immediate use. So far, Fick’s law has been typically applied even in those cases in which caution is advised.

Integrating Fick’s law across the membrane with the assumption of a uniform diffusion coefficient across the membrane yields:

(5) $$J_{i}=D_{i}\frac{∆c_{i}}{l}$$

Introducing Equation (1) into Equation (5), one finally obtains:

(6) $$J_{i}=D_{i}S_{i}\frac{∆p_{i}}{l}$$

which corresponds to Equation (1), recognizing that $P_{i}=D_{i}S_{i}$. $S_{i}$ is the incremental ratio $\frac{∆c_{i}}{∆p_{i}}$, which is a way to express the solubility of the fluid in the polymer membrane.

For high penetrant concentrations in the membranes, the assumption of a uniform diffusion coefficient is no longer valid, and an average diffusion coefficient is introduced (subscript u for the upstream side; d for the downstream side) [29]:

(7) $${\bar{D}}_{i}=\frac{1}{∆\omega_{i}}\int_{\omega_{u,i}}^{\omega_{d,i}}\frac{-D_{i}\left(\omega_{i}\right)}{1-\omega_{i}}d\omega_{i}$$

where ${\bar{D}}_{i}$ is the local diffusion coefficient, and $\omega_{i}$ is the mass fraction of the penetrant inside the polymer.

(8) $$P_{i}=S_{i}\cdot {\bar{D}}_{i}$$

Therefore, permeability can be estimated as the product of the solubility coefficient and the mutual diffusion coefficient. Consequently, high permeability can result from high solubility, high diffusivity, or a favorable combination of the two. From a phenomenological point of view, one can imagine that the permeation process consists of dissolution of the fluid inside the polymer phase, followed by diffusion across the membrane.

Usually, in permeation experiments, the downstream side is kept at low pressure, and in such conditions the selectivity of the polymer (perm selectivity) $\alpha_{i,j}$ is equal to the ratio between the permeability of the more-permeable to the less-permeable gas, and contains a solubility-selectivity ($\alpha_{i,j}^{S}$) and a diffusivity-selectivity contribution ($\alpha_{i,j}^{D}$):

(9) $$\alpha_{i,j}=\frac{P_{i}}{P_{j}}=\frac{S_{i}}{S_{j}}\cdot \frac{D_{i}}{D_{j}}=\alpha_{i,j}^{S}\cdot \alpha_{i,j}^{D}$$

Analyzing these two properties independently is a useful way to rationalize gas transport in polymers and the structure–property relationship that can guide membrane material design [30,31].

Solubility-selectivity provides an important contribution to the overall perm-selectivity in high free-volume glassy polymers, whereas for low and medium free-volume polymers, sieving effects are more important, and diffusivity-selectivity has a higher weight [32]. Some authors question the regarding of ultra-high free volume polymers, such as polymers of intrinsic microporosity (PIMs), as dense materials for which the solution–diffusion model applies; however, successful modelling studies have been performed based on this hypothesis [33]. In conclusion, the indication is that high free-volume polymeric materials can be regarded as dense as far as their separation properties are concerned if they obey the solution–diffusion model, regardless of their specific microstructure. 

An important aspect concerning estimation of selectivity and its separate contributions is the conditions at which the corresponding properties are measured: if solubility or diffusivity of pure gases are used in Equation (9), the ideal selectivity is calculated, whereas if the corresponding properties at mixed-gas conditions are used, multicomponent selectivity is obtained. 

In the membrane literature, most data refer to pure gas conditions, and only a few gas mixtures have been experimentally analyzed. Initially, the mixed-gas data were available almost exclusively for permeability [34,35,36,37]. However, over the last decade, more mixed-gas sorption and, to a more limited extent, mixed-gas diffusion studies have been performed. In the case of mixed-gas sorption, the available studies are discussed in the dedicated section, and are mostly related to CO_2_/CH_4_ and CO_2_/hydrocarbon binary mixtures and CO_2_/CH_4_/C_2_H_6_ ternary mixtures [24,29,35,36,37,38,39,40,41,42,43,44,45,46,47,48,49,50,51,52,53,54]. For mixed-gas diffusion, CO_2_/CH_4_, CO_2_/N_2_/O_2_, and CO/CO_2_/N_2_/O_2_ mixtures have been studied [55,56,57]. Such studies identified and classified differences between ideal and multicomponent selectivity, especially concerning the overall importance of the solubility-selectivity contribution [32]. Such experimental observations showed the need to develop reliable modelling tools able to predict mixed-gas behavior, possibly using only pure-gas experimental measurement as inputs in order to reduce the need for complicated and time-consuming mixed-gas tests.

Finally, it is worth noting that solubility, diffusivity, and permeability depend on temperature, pressure difference, absolute pressure, gas mixture composition, and formation history of the sample, the latter factor especially in the case of glassy polymers [19,58,59]. Therefore, any permeability or selectivity value should be coupled to this information and possibly compared with other materials at homogenous conditions. In particular, the temperature-dependence of permeability, solubility, and diffusivity is expressed by an Arrhenius law [60]:

(10) $$S=S_{0}\exp\left(-\Delta {\tilde{H}}_{s}/RT\right)$$

(11) $$D=D_{0}\exp\left(-E_{D}/RT\right)$$

(12) $$P=P_{0}\exp\left(-E_{P}/RT\right)$$

where $\Delta {\tilde{H}}_{s}$ is the molar enthalpy of sorption, and $E_{D}$ and $E_{P}$ are the activation energies of diffusion and permeation. The enthalpy of sorption for gas solubility in condensed phases (liquids or polymers) can be decomposed into two contributions [61]:

(13) $$\Delta {\tilde{H}}_{s}=\Delta {\tilde{H}}_{c}+\Delta {\tilde{H}}_{m}$$

where $\Delta {\tilde{H}}_{c}$ is the molar enthalpy of condensation of the penetrant, and $\Delta {\tilde{H}}_{m}$ is the partial molar enthalpy of mixing the condensed penetrant with the polymer segments.

## 3. Modelling the Upper Bound of Gas Separation Membranes

**Figure 1:**
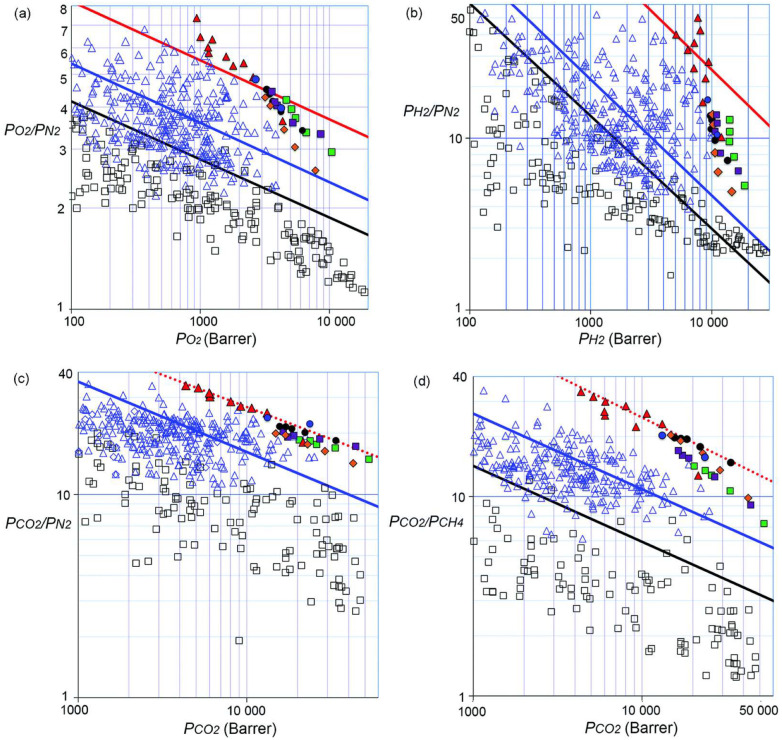
Robeson plots for the (**a**) O_2_/N_2_, (**b**) H_2_/N_2_, (**c**) CO_2_/N_2_, and (**d**) CO_2_/CH_4_ gas pairs. Upper bounds are represented by black lines (1991) and blue lines (2008). Red lines represent revisions proposed in 2015 (solid) and 2019 (dotted). Black squares are non-PIM materials, and blue triangles represent PIMs. Filled symbols represent newly synthesized ultra-permeable benzotriptycene-based PIMs. Figure reproduced from [73] under CC-BY license terms.

An important issue faced in membrane material design is the trade-off between permeability and selectivity: highly permeable materials usually display very poor selectivity, whereas highly selective materials exhibit lower permeabilities [62]. Such behavior is evidenced by several gas pairs and polymers with very different chemical natures. By reporting the logarithm of the selectivity versus the logarithm of the permeability of the most-permeable gas, polymeric membrane performance lies below a limiting line, commonly referred to as the “Robeson upper bound” [62,63]. This trade-off sets an upper limit for the selectivity that can be achieved by the membrane at a fixed permeability, and to the permeability that can be reached at a fixed selectivity [64,65]. The threshold is empirically expressed as:

(14) $$\alpha_{i,j}=\frac{\beta_{i,j}}{P_{i}^{\lambda_{i,j}}}$$

where $P_{i}$ is the permeability of the more-permeable gas, $\alpha_{i,j}$ is the selectivity of the more-permeable to the less-permeable gas, and $\beta_{i,j}$ and $\lambda_{i,j}$ are parameters specific to each gas couple. Similar trends can also be obtained for solubility and diffusivity [31]. For instance, by plotting the solubility of the more-soluble gas against solubility-selectivity, a solubility upper bound can be constructed [31]. Analogously, by plotting the diffusivity of the fastest-diffusing gas against diffusivity-selectivity, a diffusivity upper-bound is obtained [31]. Several theoretical rationalizations of these trends have been proposed, such as the use of cohesive energy density to interpret the diffusivity upper bound [66], Sanchez–Lacombe’s lattice fluid theory to interpret the solubility upper bound [67], and free-volume theory to interpret the permeability upper bound [68]. Freeman showed that the slope of the upper bound is correlated to the kinetic diameters of the gas molecules [69]:

(15) $$\lambda_{i,j}={\left(\frac{d_{k,j}}{d_{k,i}}\right)}^{2}-1$$

where $d_{k,j}$ is the kinetic diameter of the larger molecule, and $d_{k,i}$ the kinetic diameter of the smaller molecule. On the other hand, the position of the upper bound line depends both on size and solubility of the molecules:

(16) $$\beta_{i,j}=\frac{S_{i}}{S_{j}}S_{i}^{\lambda_{i,j}}\exp\left\{-\lambda_{i,j}\left[𝓫-𝓯\left(\frac{1-a}{RT}\right)\right]\right\}$$

where $S_{i}$ is the solubility coefficient of the most-permeable gas, $S_{j}$ the solubility coefficient of the less-permeable gas, and a and 𝓫 are parameters from the linear free energy relation between the preexponential factor in Arrhenius equation for diffusivity and the activation energy of diffusion observed by Barrer [70] and Van Amerongen [71]; a has a universal value of 0.64, 𝓫 is 9.2 for rubbery polymers and 11.5 for glassy ones, and 𝓯 is an adjustable universal parameter, fitted to achieve the best representation of selectivity vs. permeability data [69]. Its value has been calculated as 𝓯=12,600 cal/mol for polymers in the limiting curves drawn in 1991 and 14,154 cal/mol in the 2008 update [72]. The upper bounds for some indicative gas pairs [73] are reported in Figure 1.

Freeman showed that selectivity can be expressed as a function of these parameters as [69]:

(17) $$\ln\alpha_{i,j}=-\lambda_{i,j}\ln D_{i}+\ln\frac{S_{i}}{S_{j}}-\lambda_{i,j}\left[𝓫-𝓯\left(\frac{1-a}{RT}\right)\right]$$

Assuming that the solubility selectivity changes little with the polymer, and noting that the term $\lambda_{i,j}𝓫$ is a constant for a given gas couple and for all polymers, it follows that diffusivity plays a more important role than solubility in determining upper-bound selectivity values. This is because diffusivity values of fluids in polymers normally vary over wider ranges than solubility values, as a small size difference between permeants can result in a large diffusivity difference. The typical way to enhance the performance of glassy polymers, commonly used for gas separation, is to change the structure by introducing packing-disrupting units to increase the free volume, thus increasing the diffusion coefficient and reducing diffusivity selectivity.

However, not all separations are dominated by size selectivity: if one species is much more soluble than the other, such as in the separation of higher hydrocarbons from natural gas, volatile organic compounds from air, or CO_2_ from hydrogen, the solubility selectivity can be higher than the diffusivity selectivity, especially in rubbery polymers. These materials are called “reverse-selective”. In such cases, the performance plot does not display an upper bound, but the cloud of different material points is oriented along the opposite diagonal [74], meaning that the more permeable materials are also the more selective ones, although an upper limit in performance may be identified [75]. In such situations, low-temperature separation is preferred, as solubility is enhanced, although a specific analysis of the activation energies of the different gas mixture components should be carried out to identify the optimal temperature.

A systematic comparison of gas separation performance of glassy and rubbery polymers for several gas pairs [76] showed that glassy polymers are closer to the upper bounds for all gas pairs. This was ascribed both to a higher size-sieving ability compared to rubbery polymers and to higher solubility coefficients owing to their excess free volume. In particular, perfluorinated and partially fluorinated glassy polymers frequently exhibit the most favorable combination of permeability and perm-selectivity. An analysis of the solubility and diffusivity contributions for these important families of polymers [77] showed that solubility has a higher weight in the overall performance compared to hydrocarbon polymers.

**Figure 2:**
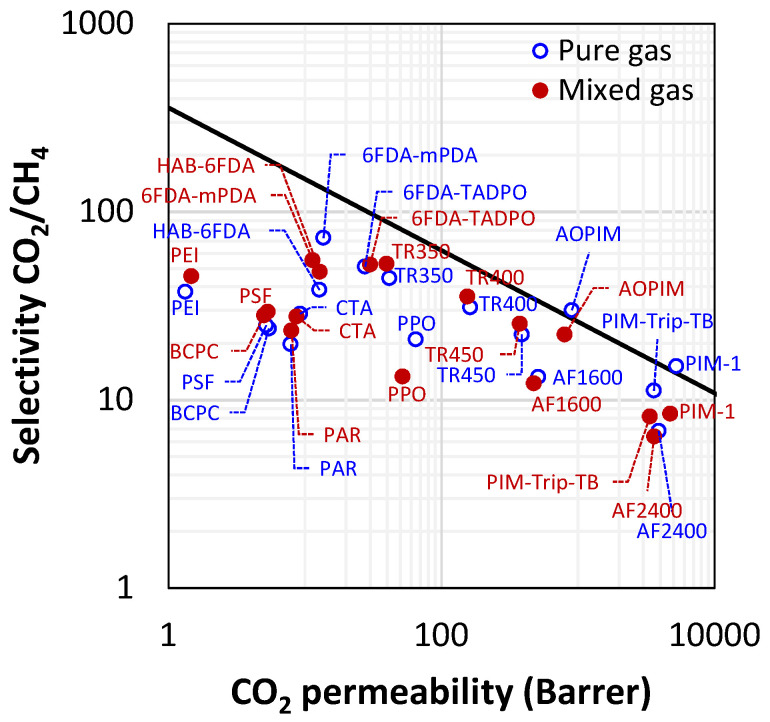
CO_2_/CH_4_ upper bound for pure-gas (empty symbols) and ~50:50 mixed-gas measurements (filled symbols) at 35 °C and 10 bar for 6FDA-TADPO [27], 6FDA-mPDA [38], PEI [81], PSF [81], CTA [82], PAR [81], PPO [83], HAB-6FDA [84], TR350 [84], TR400 [84], TR450 [84], AF1600 [85], AF2400 [85], PIM-1 [82], PIM-Trip-TB [43], and AOPIM [82].

Finally, it is noteworthy that permeability-selectivity performance plots displayed in the literature are usually obtained using pure gases at room temperature and low pressure. Studies on the effect of temperature on the position of the upper bound have been reported [78]. The effect of pressure on the position of the upper bound has been analyzed in the framework of the free-volume theory for the gas couple CO_2_/CH_4_ [79,80]. It was found that plasticization induced by high CO_2_ pressure would lower the intercept of the upper bound, $\beta_{i,j}$ [78]. Mixed-gas effects could also change the performance of membrane materials in the presence of mixtures. In particular, swelling induced by the high concentration of one gas affects the diffusivity of the other species. In glassy polymers, so-called competitive sorption limits the solubility of all species present, but to a different extent for each one. As a result, mixed-gas performance and mixed-gas upper bounds [79] significantly deviate from the ideal values obtained from pure-gas measurements. Figure 2 shows a few examples for CO_2_/CH_4_ selectivity versus CO_2_ permeability data for a series of glassy polymers suitable for separation. The blue circles refer to the estimated separation performance using pure-gas measurements, while the red ones indicate the actual performance estimated in the mixed-gas state.

## 4. Macroscopic Models for Gas Solubility in Polymers

The calculation of gas sorption in polymers consists of the solution of a phase equilibrium problem, which requires expression of the penetrant chemical potential in the polymeric phase. However, a distinction must be made based on whether the polymer is in a rubbery or glassy state. In rubbery polymers, equilibrium is reached instantaneously, such as in liquids, or within the usual experimental times, and one can choose between activity coefficient approaches or Equation-of-State (EoS) methods to calculate fluid solubility. Molecular methods have also been developed to calculate solubility and are presented and discussed later in Section 7.5. EoS models are endowed with higher predictive power and provide a complete representation of the polymer–fluid mixture. Indeed, such models allow the evaluation of the polymer–fluid mixture volume, and thus the swelling, in a predictive way. The most-employed EoS models for polymeric systems are those based on a Lattice Fluid (LF) representation of substances, such as the LF and Non-Random Hydrogen Bonding [86,87,88], and those based on hard sphere chain schemes, such as the Statistical Associating Fluid Theory (SAFT) [89]. Both approaches are very appropriate in the representation of the thermodynamic behavior of a mixture of polymer and low molecular weight species.

The case of glassy polymers is different, as the matrix is in non-equilibrium conditions, and the usual equilibrium thermodynamics results do not hold. In this case, the above-mentioned approaches cannot be applied. Calculation of gas solubility in glassy polymers is customarily performed in the literature using the empirical Dual-Mode Sorption (DMS) model [90,91,92,93,94,95,96,97,98,99,100]. Its simplicity of use and its good correlation with experimental pure-gas sorption behavior in glassy polymers favor its widespread use, mainly for data-fitting purposes. Indeed, its empirical nature makes DMS more a correlating tool, as discussed in the following sections.

A more rigorous and predictive method for glassy polymers is the Non-Equilibrium Thermodynamics for Glassy Polymers (NET-GP) approach [101]. Such methodology gives non-equilibrium expressions for the free energy of the system for any EoS of choice by introducing an internal state variable, the polymer density, to describe the out-of-equilibrium degree of the glassy mixture. This framework has been successfully applied to predict gas and vapor sorption in a variety of polymeric systems [102,103].

In the following, the theoretical foundations of the aforementioned approaches are laid out, and examples of their application to calculate mixed-gas sorption in various polymeric systems are presented.

A general overview of the models to calculate fluid solubility in polymers is given in Table 1.

### 4.1. Activity Coefficient Models

Activity coefficient models describe the non-ideality of mixtures by providing a relationship between the excess free energy of the mixture ($G^{ex}$, temperature, pressure, and composition, from which activity coefficients to be used in phase equilibrium calculations are obtained:

(18) $$\ln\gamma_{i}=\frac{\partial }{\partial n_{i}}{\left(\frac{G^{ex}}{RT}\right)}_{T,p,n_{j\neq i}}=\frac{{\bar{G}}_{i}^{ex}}{RT}$$

Activity coefficient models were originally derived for liquid mixtures and subsequently extended to encompass specific features of polymers, such as high molecular weight, but also free volume, crosslinks, or semicrystalline structure.

**Figure 3:**
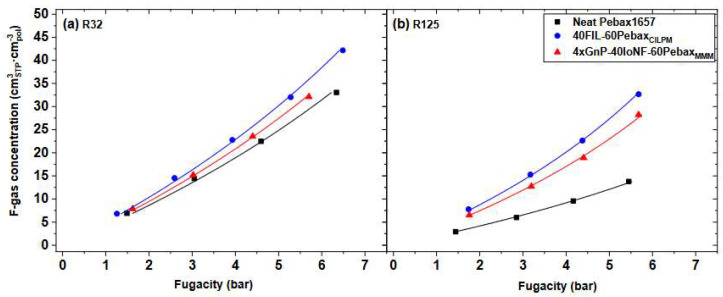
Sorption isotherms of (**a**) R32 (difluoromethane) and (**b**) R125 (pentafluoroethane) at 30 °C in a Pebax membrane and in two mixed-matrix membranes containing ionic liquids. Solid lines correspond to the fit of experimental data to the Flory–Huggins model with varying values of the interaction parameter $\chi_{ij}$. Figure reproduced from [116] under CC-BY license terms.

The most important activity coefficient model used for polymer solubility is the Flory–Huggins one [106,107], which was developed to describe the Gibbs free energy of mixing polymeric mixtures using statistical concepts for the mixing entropy, by invoking, for the first time, the idea of a lattice to describe matter. The model can be used to describe the behavior of amorphous rubbery polymers, and extensions exist to account for elastic contributions due to crosslinking [108] or for the presence of a crystalline fraction in the polymer [109]. Other activity coefficient models such as Non-Random Two Liquids (NRTL) [110,111] or UNIFAC [112,113] have been modified and tested for the calculation of solubility in polymers, obtaining different results. The review by Lipnizki and Tragard [114] provides examples of application to membrane separation of many activity-coefficients models. Recent applications of the Flory–Huggins and NRTL models to membrane systems can be found in [115,116,117], and an example is shown in Figure 3.

One important limitation encountered in the application of this class of models to gas sorption is that they do not provide a relation between density, temperature, pressure, and composition (i.e., an equation of state); therefore, they cannot describe polymer swelling during sorption.

### 4.2. EoS Models: Lattice Fluid Equations of State

Lattice fluid (LF) theories employ statistical mechanics arguments to derive expressions for the free energy of the system G and, in turn, of all other thermodynamic properties of the system, including the chemical potential, according to its definition:

(19) $$\mu_{i}={\left(\frac{\partial G}{\partial n_{i}}\right)}_{T,p,n_{j\neq i}}$$

In the lattice-fluid representation, each molecule is considered a flexible chain composed of r segments (*mers*) immersed in a lattice of cubic cells. The Flory–Huggins model [106,107] assumes the lattice to be fully occupied, while in the Sanchez and Lacombe model [86,104], empty cells are possible in the system. The entropy of the system is estimated through the number of possible configurations of the lattice. The energy of the lattice is obtained by summing all the pairwise energetic contributions of first neighbors and considering null interaction between molecule segments and empty cells. The Gibbs free energy expression in this model thus becomes:

(20) $$G=Nrk_{B}T^{*}\left\{-\tilde{\rho }+\frac{\tilde{p}}{\tilde{\rho }}+\tilde{T}\left[\frac{1-\tilde{\rho }}{\tilde{\rho }}\ln\left(1-\tilde{\rho }\right)+\frac{1}{r}\ln\left(\tilde{\rho }\right)\right]\right\}$$

where $\tilde{T}$, $\tilde{p}$, and $\tilde{\rho }$ are the reduced temperature, pressure, and density, respectively, defined in Appendix B. Each substance is univocally characterized by the macroscopic parameters $T^{*}$, $p^{*}$, and $\rho^{*}$, which are related by the relations reported in Appendix B. The characteristic pressure of the system, $p^{*}$, is associated with its cohesive energy density, i.e., the strength of intermolecular interactions.

By minimizing the free energy with respect to volume at a constant temperature and pressure, one obtains the Lattice Fluid EoS, which is formally identical for pure components and mixtures, provided that the corresponding definition of the reduced variables $\tilde{T,}\;\tilde{p},\;\tilde{\rho }$ is used:

(21) $$\tilde{\rho }=1-\exp\left[-\frac{{\tilde{\rho }}^{2}}{\tilde{T}}-\frac{\tilde{p}}{\tilde{T}}-\tilde{\rho }\left(1-\sum_{i}^{N}\frac{\phi_{i}}{r_{i}}\right)\right]$$

Therefore, the extension to mixtures is straightforward. Each species present in the mixture occupies $N_{r,i}r_{i}$ lattice cells, and the composition of the system $\phi_{i}$ is expressed as the fraction of lattice sites occupied by i. Furthermore, it is assumed that the close-packed volume of each species is conserved at multicomponent conditions, and the total number of binary interactions in the mixture is the sum of the corresponding interactions for the pure components. These two hypotheses grant additivity of the close-packed volumes.

Mixing rules for the macroscopic parameters are:

(22) $$\frac{1}{\rho^{*}}=\sum_{i}^{N}\frac{\omega_{i}}{\rho_{i}^{*}}$$

(23) $$p^{*}=\sum_{i}^{N}\phi_{i}p_{i}^{*}-\sum_{i}^{N-1}\sum_{j>i}^{N}\phi_{i}\phi_{j}\Delta p_{ij}^{*}\;\mathrm{where}\;∆p_{ij}^{*}=p_{i}^{*}+p_{j}^{*}-2\left(1-k_{ij}\right)\sqrt{p_{i}^{*}\cdot p_{j}^{*}}$$

(24) $$\tilde{\rho }=1-\exp\left[-\frac{{\tilde{\rho }}^{2}}{\tilde{T}}-\frac{\tilde{p}}{\tilde{T}}-\tilde{\rho }\left(1-\sum_{i}^{N}\frac{\phi_{i}}{r_{i}}\right)\right]$$

where $∆p_{ij}^{*}$ expresses the characteristic binary interactions between species *i* and *j* and contains an adjustable parameter $k_{ij}$, to account for deviations from the geometric mean mixing rule. Such a parameter is present in practically every EoS model.

**Figure 4:**
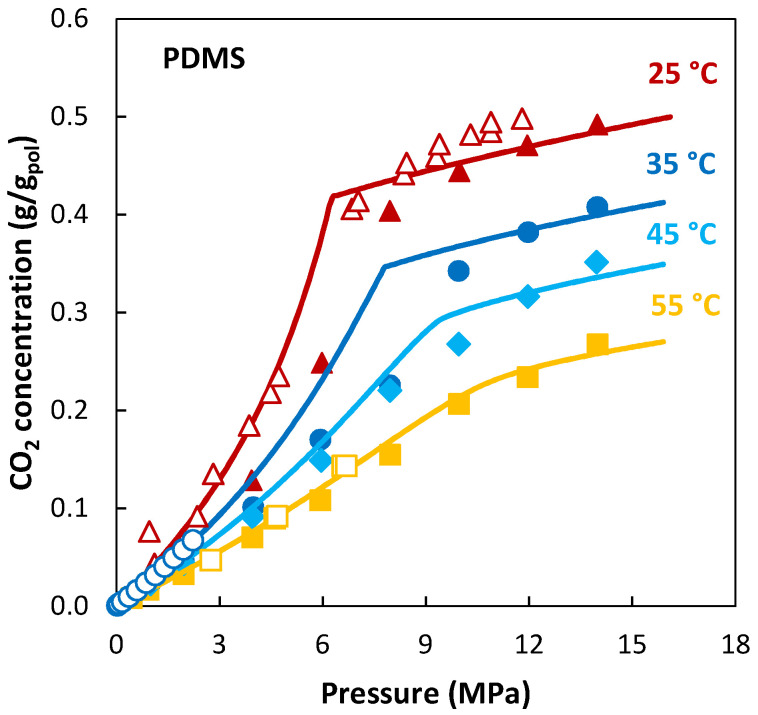
CO_2_ sorption in PDMS at different temperatures. Filled symbols: experimental data from [122]: triangles, 25 °C; circles, 35 °C; diamonds, 45 °C; squares, 55 °C. Empty triangles: experimental data from [123] (25 °C). Empty circles: experimental data from [124] (35 °C). Empty squares: experimental data from [125] (50 °C). Lines: LF EoS calculations. Reproduced with permission from [121]. Copyright 2022, Elsevier.

Recent studies employing the Sanchez–Lacombe EoS in the study of gas/polymer systems can be found in [118,119,120,121]. Figure 4 is a representation of one the latest applications of the theory to the sorption of pure CO_2_ in polydimethylsiloxane (PDMS), a rubbery membrane. The pressure range considered in the measurements encompasses the transition from gas-like to liquid-like behavior of CO_2_, which is reflected in the sorption trend by a reduction of the slope. The LF model is able to represent such a transition in the sorption trend at each temperature correctly without adding any adjustable parameter.

Another model belonging to the class of compressible lattice theories, but accounting for specific polar interactions between sites, is the Non-random Hydrogen Bonding (NRHB) theory [87,88]. This model contains parameters for pure components plus additional parameters for associating interactions. The first two pure component parameters are the enthalpic and entropic contributions, $\varepsilon_{i,h}^{*}$ and $\varepsilon_{i,s}^{*}$, respectively, to the mean interaction energy per molar segment $\varepsilon_{i}^{*}$, correlated through the following equation:

(25) $$\varepsilon_{i}^{*}=\varepsilon_{i,h}^{*}+\left(T-298.15\right)\varepsilon_{i,s}^{*}$$

The third parameter is associated with the close-packed density of the lattice $\rho_{i}^{*}$. As in the lattice fluid theory, the first three parameters are usually fitted on LV equilibrium data for the fluids and on PVT data for the polymers. The fourth parameter associated with each component i is the shape factor, $s_{i}$, which represents the ratio of molar surface to molar volume, and it is usually estimated via the group contribution UNIFAC [126,127].

In this model, the binary interaction parameter $k_{ij}$ acts on the characteristic energy rather than on the characteristic pressure as in the LF model:

(26) $$\varepsilon_{ij}^{*}=\left(1-k_{ij}\right)\sqrt{\varepsilon_{i}^{*}\varepsilon_{j}^{*}}$$

For systems displaying hydrogen bonding or Lewis acid/Lewis base interactions, two additional parameters for each association interaction between a functional group α and a functional group β are introduced: the association energy $E_{\alpha \beta }^{0}$ and the association entropy $S_{\alpha \beta }^{0}$. The values of the association parameters relative to a given component (self-association) can be retrieved by fitting the equilibrium thermophysical properties of the species. Cross-interaction parameters between functional groups belonging to different molecules are estimated via combining rules of the two self-associating parameters:

(27) $$E_{\alpha \beta }^{0}=\frac{E_{\alpha \alpha }^{0}+E_{\beta \beta }^{0}}{2};S_{\alpha \beta }^{0}={\left(\frac{{S_{\alpha \alpha }^{0}}^{1/3}+{S_{\beta \beta }^{0}}^{1/3}}{2}\right)}^{1/3}$$

For the expressions of chemical potential, we direct the reader to the original papers [87,88].

### 4.3. EoS Models: Statistical Associating Fluid Theory (SAFT)

**Figure 5:**
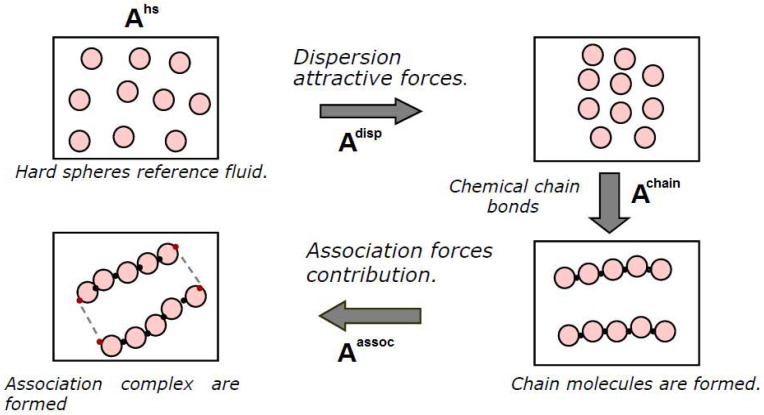
Schematic of different contribution to Helmholtz free energy in SAFT theory. Reproduced with permission from [136]. Copyright 2015, Elsevier.

Equations of state based on Statistical Associating Fluid Theory are a family of models that possess a strong theoretical foundation based on molecular considerations. The SAFT models were initially developed in the early 1990s [128,129] and have undergone numerous subsequent modifications [130,131]. They are all based on Wertheim’s perturbation theory [132,133,134] and thus belong to the so-called “perturbative models”. Perturbative methods start by providing an expression for the thermodynamic properties of a reference fluid. The thermodynamic properties of all other systems can then be calculated using additional contributions (perturbations) that account for deviations from the reference fluid. Such contributions can be given by rigorous equations, polynomial expansions, or empirical terms. SAFT models usually provide the expression for the residual Helmholtz free energy $A^{res}$, i.e., the difference between the actual Helmholtz free energy and that of the ideal gas at the same temperature and volume. One possible example of such methodology, corresponding to the picture shown in Figure 5, is reported below:

(28) $$A^{res}=A^{hs}+A^{disp}+A^{chain}+A^{assoc}$$

The different terms refer to interaction terms of the real fluid:

- Hard sphere repulsive interaction (*hs*), which is a property of the reference fluid;
- Attractive dispersion terms (*disp*), corresponding to the formation of weak interactions;
- Chain formation contribution (*chain*), relative to the formation of covalent bonds;
- Association interaction contributions (*assoc*), for polar interactions between groups.

The difference between the various SAFT versions is related to the different expressions used to calculate the various terms and for the “reference fluid” chosen. For instance, in the Huang and Radosz version (HR-SAFT) [129], the dispersion term is based on a square well approximation of the binary interaction potential fitted on the data for Argon, while in the Perturbed Chain SAFT (PC-SAFT) proposed by Sadowski et al. [135], the perturbations are applied to a hard chain system and the model is able to more accurately represent the chain-like shape of linear alkanes and polymers.

Pure fluid properties are described with three parameters: the radius *σ* of the sphere, the number of spheres per molecule *m*, and the interaction energy *u_0_*, which relates well to the depth of potential energy. Mixtures usually require an additional binary parameter for couples of species in the mixture, which affects the interaction energy parameters. At least two associating parameters are required for each hydrogen bonding interaction. Such parameters refer to the interaction energy and the distance between associating groups.

The chemical potential required for calculating the solubility of the fluid in the mixture is obtained as:

(29) $$\mu_{i}-\mu_{i}^{IG}=\frac{\partial }{\partial n_{i}}{\left(\frac{A^{res}}{RT}\right)}_{T,V,n_{j\neq i}}$$

For equilibrium calculations, the chemical potential of the different components in the vapor phase and in the polymer phase have to be equal so that for every vapor mixture composition and set of operative conditions, a system in N_c_ − 1 equations with N_c_ − 1 unknowns can be written, which can be solved in order to obtain the equilibrium concentration of different penetrants in the polymer. The general expression of the SAFT EoS is not simple and usually cannot be written in a single equation. The set of equations to be solved for solubility calculation in the case of PC-SAFT is reported in the Appendix of [135].

**Figure 6:**
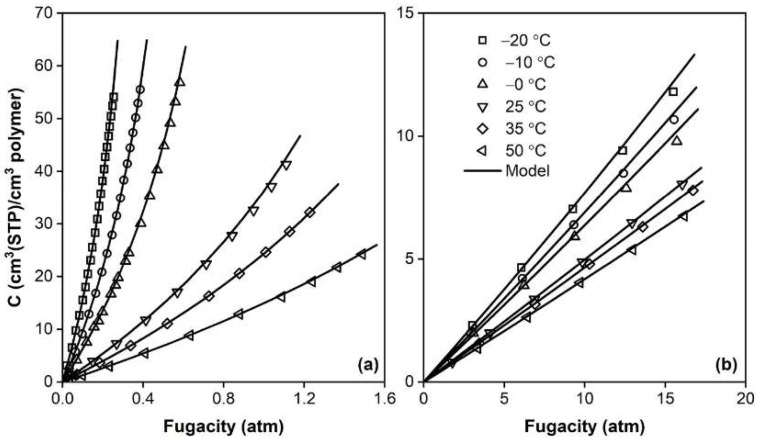
Example application of the PC-SAFT model to calculate (**a**) n-C_4_H_10_ and (**b**) CH_4_ sorption isotherms as a function of temperature [137]. Experimental data from [47]. Reprinted with permission from [137]. Copyright 2020, Elsevier.

The application of different type of SAFT models to phase equilibria involving polymers has been considered in several works, considering amorphous and crystalline systems, as well as random and block-copolymers [130], and has continued to be routinely applied in recent studies [137,138,139,140] with success, as shown, for example, in Figure 6. However, due to the inherent complexity of the EoS, SAFT models are seldom employed in the analysis of penetrant permeability, although some examples may be found in the literature [137,141].

### 4.4. Non-Equilibrium Thermodynamics for Glassy Polymers (NET-GP)

The Non-Equilibrium Thermodynamics for Glassy Polymers (NET-GP) approach [101,102,142] provides an extension of EoS theories to non-equilibrium materials, and is therefore suitable for the calculation of the solubility of fluids in glassy polymers. The NET-GP approach applies to homogeneous, isotropic, and amorphous phases. The non-equilibrium density of the glassy polymer *ρ_pol_* acts as an internal state variable and accounts for the out of equilibrium degree of the material. The theory provides a method to calculate the non-equilibrium chemical potential by using the free energy expression provided by any EoS:

(30) $$\mu_{i}^{NE}\left(T,p,\bar{\omega },\rho_{pol}\right)=\mu_{i}^{Eq}\left(T,\bar{\omega },\rho_{pol}\right)$$

Even though the glassy polymer is not in a thermodynamic equilibrium state because it tends to densify over time, this process is slow compared to the characteristic time of a sorption process; therefore, it is possible to assume that a “pseudo” phase equilibrium condition can be reached by the polymer in contact with the gas phase, and thus calculate the amount of sorbed gas by imposing the equality of the chemical potential of the penetrant in the two phases:

(31) $$\mu_{i}^{NE\left(pol\right)}\left(T,p,\bar{\omega },\rho_{pol}\right)=\mu_{i}^{Eq\left(gas\right)}\left(T,p,\bar{y}\right)$$

The equilibrium chemical potential in the gas phase $\mu_{i}^{Eq\left(gas\right)}$ is obtained by means of a suitable equation of state for the gas phase.

The NET-GP approach requires knowledge of the polymer density at each pressure used in the computation of the sorption isotherm. For the proper evaluation of its value during sorption, experimental dilation measurements are needed. However, when such data are lacking, a linear relation between polymer specific volume and partial pressure of each penetrant can be assumed, as this has often been observed experimentally for different light gases [58,124,143]. At these conditions, adjustable swelling coefficients *k_sw,i_* can be defined as follows:

(32) $$\frac{1}{\rho_{pol}}=\frac{1}{\rho_{pol}^{0}}\left(1+\overset{N_{p}}{\underset{i=1}{\sum }}k_{sw,i}p_{i}\right)$$

In the case of a single penetrant, $k_{sw}$ can be evaluated by knowledge of one point of the sorption isotherm in the high-pressure range. When $T_{g}$ is experimentally accessible, $k_{sw}$ values can also be predicted by using the rheology model presented in [144]. Hasani et al. [145] recently proposed a predictive sorption calculation framework, using the latter formulation for the swelling calculation [144] and estimating the binary coefficients $k_{ij}$ independently by considering an empirical correlation between $k_{ij}$ and the Hansen solubility parameters for several polymers.

Shoghl and coworkers [146] bypassed the need for the swelling coefficient by introducing an estimate of the polymer free volume as a function of solute concentration, dry polymer density, and the lattice fluid characteristic density $\rho^{*}$. The approach provides accurate results in the case of non-swelling gases, such as CH_4_, N_2_, and Ar, for which the effect of the free-volume correction is expected to be modest. In the case of a swelling agent, such as CO_2_, the prediction is still in fairly reasonable agreement with the data; however, the model does not seem to correctly capture the shape of the sorption isotherms in the materials analyzed.

Recently, Marshall et al. [147] proposed the dry glass reference perturbation theory (DGRPT) to predict polymer swelling within the NETGP framework once the density of the pure (unpenetrated) polymer $\rho_{pol}^{0}$ is known, thus reducing the number of adjustable parameters and/or experimental data required to compute solubility at high pressure. This method provides a closure relation for the polymer chemical potential through perturbation of the dry glassy reference state, allowing the self-consistent calculation of the swollen polymer density in the presence of pure gases or mixtures. The approach yielded good results in the calculation of sorption of pure and binary vapors and liquids in various glassy polymers [147].

The most popular application of the NET-GP theory makes use of the Lattice Fluid Equation-of-State frame-of-reference, and goes by the name of Non-Equilibrium Lattice Fluid (NELF) model [101,102,142,148], which is the extension of the Sanchez–Lacombe (SL) LF EoS [86,104,149] to the non-equilibrium glassy state by means of the NET-GP theory.

As previously mentioned, in the non-equilibrium phase, the polymer density value, needed to calculate the parameters, must be determined experimentally, whereas for the gas phase, the equilibrium density results from solving the SL LF EoS.

The expression of the chemical potential of the SL LF model, to be used in Equation (31), is given below.

(33) $$\frac{\mu_{i}}{RT}=ln\left(\tilde{\rho }\phi_{i}\right)-\ln\left(1-\tilde{\rho }\right)\left[r_{i}^{0}+\frac{r_{i}+r_{i}^{0}}{\tilde{\rho }}\right]-r_{i}-\tilde{\rho }\frac{r_{i}^{0}\nu_{i}^{*}}{RT}\left[p_{i}^{*}+\overset{N}{\underset{j=1}{\sum }}\phi_{i}\left(p_{j}^{*}-∆p_{i,j}^{*}\right)\right]+1$$

Definitions of the variables used are reported in Appendix B.

Some studies that have applied the NELF model in recent years have tested the model in a variety of systems and conditions, such as commodity polymers and high performance ones, both at low and high pressure, for light gases and condensable vapors, and with mixed-matrix membranes and semicrystalline materials [121,150,151,152,153,154].

The NE approach can be applied to the SAFT and PC-SAFT EoS as well, yielding the NE-SAFT and NE-PC-SAFT models, respectively [155], which are better-suited to describe polar and associating species, but no explicit expression is available for the chemical potential due to the higher complexity of the free energy expression [156]. Application of the NET-GP framework to the Non-Random Hydrogen Bonding (NRHB) EoS [87,88] has been reported as well [157,158], also in the mixed-gas case [159,160].

### 4.5. Dual-Mode Sorption (DMS) Model for Glassy Polymers

The Dual-Mode Sorption (DMS) model [90,91,92,93,94,95,96,97,98,99,100] postulates the existence of two different gas populations at equilibrium with one another inside glassy polymers,. The first one is dissolved in the dense portion of the material, and it is described by Henry’s law. The second one saturates the non-equilibrium excess free volume of the polymer, thought of as microvoids in the polymer phase, and it is described by a Langmuir curve. The total sorbed gas as a function of gas fugacity can be expressed as a sum of these two contributions [92,98]:

(34) $$c_{i}=k_{D,i}f_{i}+\frac{C_{H,i}^{\prime }b_{i}f_{i}}{1+b_{i}f_{i}}$$

The parameter $k_{D,i}$ is Henry’s law constant, while $b_{i}$ is the Langmuir affinity constant, which represents the ratio of the rate constants of sorption and desorption of penetrants in the microvoids. $C_{H,i}^{\prime }$ is the Langmuir capacity constant, which characterizes the sorption capacity of a glassy polymer for a certain penetrant in the low-pressure region and is connected to the excess free volume, which can vary with sample history [161,162]. For every gas–polymer pair, the three parameters are retrieved through a nonlinear least-square best-fit of pure-gas sorption data. $C_{H,i}^{\prime }$ decreases as temperature increases and has been shown to vanish at the glass transition temperature ($T_{g}$) of the polymer [163], while the temperature dependence of $k_{D}$ and b is described by a van’t Hoff relation [61]:

(35) $$k_{D}=k_{D0}e^{-\frac{∆{\tilde{H}}_{D}}{RT}}$$

(36) $$b=b_{0}e^{-\frac{∆{\tilde{H}}_{b}}{RT}}$$

In Equations (35) and (36), $∆{\tilde{H}}_{D}$ and $∆{\tilde{H}}_{b}$ are the enthalpies of sorption for Henry and Langmuir modes, respectively, R is the gas constant, and T is the temperature.

Extension of this model to multicomponent sorption [164] is based on phenomenological arguments, suggested by the theory of competitive sorption of gases on catalysts, which exhibit a Langmuir behavior. The amount of free volume in a polymer is limited, because the model does not consider swelling; therefore, the various penetrants compete to occupy it, and, as a consequence, the sorbed concentration will be lower than in the pure-gas case. It is assumed that the competition is controlled by the relative values of the product of the affinity constant and partial pressure (or fugacity) of each penetrant. Further hypotheses are that the affinity parameter $b_{i}$, Henry’s constant $k_{D,i}$, and the molar density of a component sorbed inside the Langmuir sites are independent of the presence of other penetrants. The final expression for the concentration of component *i* in the presence of a second component *j* is given by:

(37) $$c_{i}=k_{D,i}f_{i}+\frac{C_{H,i}^{\prime }b_{i}f_{i}}{1+b_{i}f_{i}+b_{j}f_{j}}$$

In the case of more than 2 penetrants, the general expression is:

(38) $$c_{i}=k_{D,i}f_{i}+\frac{C_{H,i}^{\prime }b_{i}f_{i}}{1+b_{i}f_{i}+\sum_{j\neq i}b_{j}f_{j}}$$

The characteristic gas–polymer parameters found in Equations (37) and (38) are the same as those in Equation (34), which are retrieved from a least-square fit of pure gas isotherms. It is also commonplace to write Equations (34) and (37) using the partial pressure of each gas instead of its fugacity. However, when the approximation of ideal-gas behavior is not valid, such as when high pressures or gas mixtures are considered, the fugacity constitutes a more appropriate measure of the chemical potential, which is the driving force for gas sorption in the polymer. It has been verified that using pressure-based or fugacity-based parameters yields the same results in mixed-gas sorption calculations; therefore, the accuracy of the multicomponent calculations with the DMS model does not depend on this choice [46,49,51,165].

The DMS model correlates the pure sorption isotherms of most penetrants in glassy polymers well; however, it does not allow representation of all types of isotherms encountered, such as the sigmoidal ones of alcohols in glassy polymers [166]. There have been studies aimed at overcoming this limitation: for example, by incorporating multilayer sorption theory, a DMS based model capable of representing all the different shapes of sorption isotherms encountered was developed [167]. Another issue is that the adjustable parameters of the DMS model depend on polymer history and operating conditions, as well as on the temperature and pressure range investigated, and they lack predictive ability outside their range of derivation [168].

Furthermore, some inconsistencies are intrinsically related to the main assumptions of the model: the approach does not explicitly account for penetrant-induced swelling even though it may be associated with the physical dissolution mechanism, as no change in the Langmuir capacity (and thus in the excess free volume contribution) is considered in the whole solubility isotherm. When the sorption/desorption hysteresis is then inspected (see e.g., [169]), different $C_{H}^{\prime }$ values are required to describe the two different branches (pressure increasing or pressure decreasing), thus leading to a physical inconsistency of a model parameter, the Langmuir capacity, which undergoes a step-change corresponding to the maximum pressure data point, and, as such, it assumes two different values at the same point. These model parameters do not actually represent material properties, and they should rather be considered as coefficients of a useful mathematical equation able to represent some types of solubility isotherms in glassy polymers [170].

Also the multicomponent version of the DMS model does not explicitly account for the fact that the polymer matrix can swell when sorbing penetrants, and possible synergistic effects are thus not represented. Although swelling effects are negligible with respect to competition ones in many systems, such as the ultra-high free volume polymers of intrinsic microporosity (PIMs) [39,40,42,53], where the DMS model is expected to reliably estimate data [51], a detailed analysis of the multicomponent performance of the DMS model pointed out a fundamental lack of robustness [42,165]. Analysis of mixed CO_2_/CH_4_ sorption in high free volume glassy polymers indeed revealed that pure gas solubility can be represented with the same accuracy by several different DMS parameter sets, which, however, yield markedly different mixed-gas predictions that are not always accurate.

The Dual-Mode Sorption theory is applied in the development of the Partial Immobilization Dual-Mobility Model for Permeability, which is described in Section 6.1.

### 4.6. Guggenheim−Anderson−de Boer (GAB) Model

An alternative description for sorption of gases in polymers is provided by the Guggenheim–Anderson–de Boer (GAB) model [171,172,173], in which the polymer chains are considered as solids surrounded by void pockets, and the penetrant molecules are assumed to only adsorb on the polymer surfaces. This is an extension of the Brunauer−Emmett−Teller (BET) approach to multilayer adsorption of small molecules in a solid adsorbent [174,175]. This approach has proven effective for describing solubility isotherms in rubbery phases and glassy phases [167,176,177,178].

The GAB model considers a multilayer adsorption mechanism on top of the first adsorption monolayer with capacity $v_{m}$. The binding of the first monolayer on the pore walls is assumed to be stronger than that of the subsequent layers, which is expressed through a dimensionless factor h. For a single gas, the sorption isotherm has the following expression [176]:

(39) $$v=\frac{v_{m}hp^{*}p}{\left(p^{*}-p\right)\left(hp^{*}+p^{*}-p\right)}$$

where v is the penetrant sorbed mass ratio, p is the penetrant pressure, and $p^{*}$ is a reference pressure value associated to the penetrant. Therefore, the model contains three adjustable parameters for each penetrant−polymer pair, namely, $v_{m}$, h, and $p^{*}$, which are obtained as a best-fit of the experimental sorption isotherms. The dimensionless factor h is considered independent of temperature, while $v_{m}$ and $p^{*}$ are expected to follow a van’t Hoff temperature dependence.

The GAB model isotherm is more flexible than the DMS one, and it is able to represent different types of isotherms, both with concavity toward the pressure axis or toward the concentration axis, as well as S-shaped isotherms in which the initial concavity toward the pressure axis turns toward the concentration axis at higher pressures. With the addition of a concentration dependence of the parameter h, it is also possible to represent sorption isotherms in which initial concavity toward the concentration axis later turns into concavity to the pressure axis, such as those shown by alcohols in some glassy polymers [179].

To perform mixed-gas sorption calculations, it is assumed that each penetrant can sorb either on the sorption centers of the polymer or on the sorption centers created by the molecules of another compound sorbed in the polymer [180]. This leads to isotherm expressions given by the sum of different contributions, as follows:

(40) $$v_{i}=\frac{v_{m,i}h_{i}p_{i}^{*}p}{\left(p_{i}^{*}-p\right)\left(h_{i}p_{i}^{*}+p_{i}^{*}-p\right)}+\frac{r_{ij}v_{j}h_{ij}p_{i}^{*}p}{\left(p_{i}^{*}-p\right)\left(h_{ij}p_{i}^{*}+p_{i}^{*}-p\right)}$$

The first term in Equation (40) contains only parameters associated with species i, which are obtained by the best-fit of the pure component sorption isotherm; however, the second term contains two parameters related to penetrant−penetrant interactions, $r_{ij}$ and $h_{ij}$. Similarly, for species j, two further parameters are required, $r_{ji}$ and $h_{ji}$. These parameters must be obtained from the best-fit of mixed-gas sorption isotherms.

A comparative study [170] showed that increasing the number of adjustable parameters does not yield significant benefits in terms of accuracy of mixed-gas sorption calculations, with the important drawback that, unlike the two aforementioned approaches, it cannot be used predictively. Therefore, in the following section, where an example of application of modelling tools for mixed-gas sorption is presented, the GAB model is not considered.

### 4.7. Fractal Model for Solubility Coefficients

A less-adopted approach to describe solubility coefficients (and diffusivity, see Section 5.2) leverages fractal theory concepts. Mathematically, fractals are self-similar objects that show no variations regarding local dilatation. Fractal objects are described through three-dimensional parameters related to: the dimension of Euclidean space d, which is the fundamental space of classical geometry; the fractal object dimension $d_{f}$, which describes the object density reduction gradient; and the spectral fraction dimension $d_{s}$, which depicts the object connectivity [181,182]. It has been suggested that thermodynamically non-equilibrium solids, such as glassy polymers, can be treated as fractal objects [182,183], and solubility coefficients in agreement with experimental results have been calculated using this theoretical framework [184,185].

In this model, it is assumed that small gas molecules are non-interacting with the polymer chains, that they are adsorbed on the walls of free volume microvoids, and that gas–gas molecular interactions can be characterized using the Lennard–Jones potential $\varepsilon /k_{B}$. In this case, the fractal equation for calculating the solubility coefficient S may be written as [186]:

(41) $$S=S_{0}{\left(F_{g}^{ef}\right)}^{D_{f}/2}\left(\frac{\varepsilon }{k_{B}}\right)$$

where $F_{g}^{ef}$ is the effective cross-sectional area of the sorbed gas molecules averaged over all possible orientations by considering maximum and minimum diameters of the gas molecules to estimate its effective diameter [182,184]; $D_{f}$ is the global fractal dimension parameter, which can be calculated by a series of correlations, as detailed in the following [182,183].

$S_{0}$ corresponds to a minimum solubility of a gas molecule where a gas molecular interaction does not count. The estimated value of $S_{0}$ for PVTMS has been reported as 4.0 × 10^−8^ [184].

The relative fraction of the closely packed segments in clusters ($\varphi_{cl}$) is introduced and calculated from knowledge of the glass transition temperature of the polymer ($T_{g}$) through the following percolation correlation:

(42) $$\varphi_{cl}=0.03\left(1-X_{cr}\right){\left(T_{g}-T\right)}^{0.55}$$

where T is the temperature at which the parameter is measured, e.g., 293 K, and $X_{\mathrm{cr}}$ is the degree of crystallinity, which, for many glassy polymers, is near zero.

The fractal dimension of the polymer structure, $d_{f}$, is calculated employing the following equation [33]:

(43) $$d_{f}=3-6{\left(\frac{\varphi_{cl}}{C_{S}\;A_{cr}}\right)}^{1/2}$$

where $A_{cr}$ is the cross-sectional area of a macromolecule in Å^2^, and $C_{S}$ is a characteristic ratio that represents the index of chain flexibility [187,188]. Ways of estimating the values of $A_{cr}$ and $C_{S}$ have been reported in the literature [186,188,189].

Finally, the global fractal dimension, $D_{f}$, can be obtained from the flowing equation:

(44) $$D_{f}=1+\frac{1}{3-d_{f}}$$

This method has been applied to calculate the solubility coefficients of hydrogen, nitrogen, oxygen, carbon dioxide, methane, ethylene, and propylene in polynorbornenes [184,185], achieving predictions on average within 30% of the experimental results, except for CO_2_, which was underpredicted by approximately a factor of six.

The model has not yet been extended to the mixed-gas case. Moreover, one drawback of its application to innovative glassy polymer membranes is that it requires knowledge of the $T_{g}$ of the polymer, which, for a large number of these materials, is not known experimentally.

### 4.8. Insight on Mixed-Gas Sorption in Polymers: Experimental Trends and Modeling

The sorption of gas mixtures in polymer membranes has shown that they exhibit rather marked deviation from ideal pure-gas behavior. Considering, for simplicity, binary mixtures, in glassy polymers, due to competitive sorption effects, the solubility of both species at mixed-gas conditions is generally lower than the corresponding pure-gas solubility at the same gas fugacity. However, it has been observed that the effect on solubility-selectivity depends on the relative amounts of the two species absorbed in the polymer. Solubility-selectivity can be calculated by making use of the definition of the solubility coefficient S using the corresponding value of the gas concentrations c at pure- or mixed-gas conditions.

(45) $$\alpha_{A,B}^{S}=\frac{S_{A}}{S_{B}}=\frac{c_{A}/f_{A}}{c_{B}/f_{B}}$$

For instance, in the case of a CO_2_/CH_4_ mixture in many glassy polymers, CO_2_ is usually the most abundant component in the polymer, and the multicomponent CO_2_/CH_4_ solubility-selectivity is higher than the “ideal” value calculated considering the pure-gas solubility ratio of the two gases at the same fugacity. However, there is a range of conditions when the gas mixture is extremely rich in CH_4_ and poor in CO_2_, in which there are more CH_4_ than CO_2_ molecules sorbed in the polymer. In such cases, it has been observed that the CO_2_/CH_4_ solubility-selectivity is lower than the “ideal” value. Such approximate correlation of the solubility-selectivity changing with respect to the relative amount of the sorbed gases [41,190] has been found to hold true for several mixtures in glassy polymers, collected in Table 2. In Table 2, “Competition” effects associated with sorption indicate that the solubility of one or both gases is lower than the corresponding pure-gas value at the same fugacity in the composition range inspected in the tests. This is associated with an increase in the solubility selectivity and this is the prevalent phenomenon observed for glassy polymers. The term “Swelling” indicates that the solubility of one or both gases is higher than the corresponding pure-gas value at the same fugacity, with a detrimental effect on solubility-selectivity. In rubbery polymers, the swelling effects are usually dominant.

In all cases, these effects need to be accounted for in the design of the separation operation, in order to avoid significant errors in membrane performance estimation. Mixed-gas experiments are very delicate and much more time-consuming than pure-gas tests. Therefore, there is the clear need for reliable models that involve a minimum number of adjustable parameters.

As an example, we show the case of PIM-1: CO_2_/CH_4_ mixed-gas sorption has been characterized thoroughly in this polymer, and the NELF model parameters are available in the literature. The NELF parameters for PIM-1 were retrieved by analyzing a large dataset comprising solubility at infinite dilution of light gases and several vapors [33]. Mixed-gas sorption calculations with the NELF model also require the use of a binary interaction parameter for the gas couple, which can be optimized by fitting equilibrium data for the gas mixture with the corresponding equilibrium model (the SL LF EoS). However, the effect of this parameter on mixed-gas sorption results has been found to be negligible in most cases [32,41,190].

#### Modelling Mixed-Gas Sorption of CO_2_/CH_4_ Mixture in PIM-1: NELF and DMS Model Results

**Figure 7:**
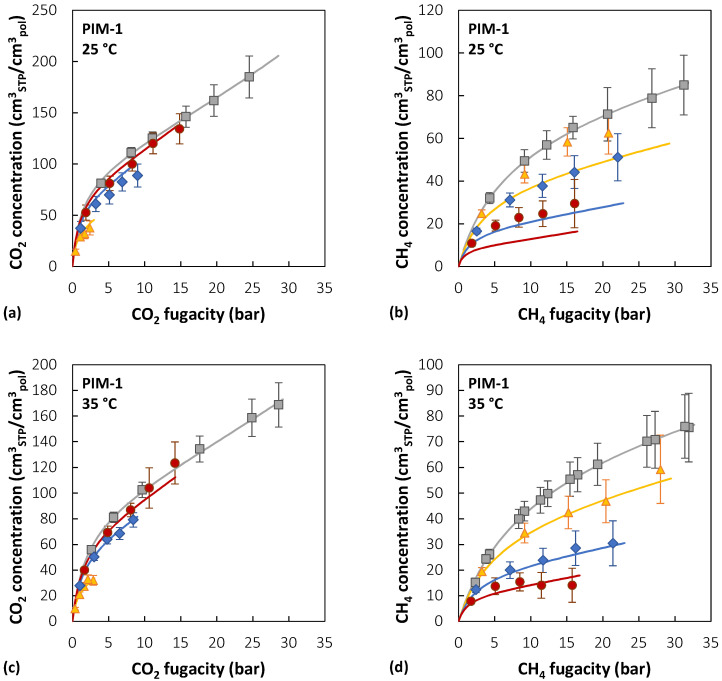

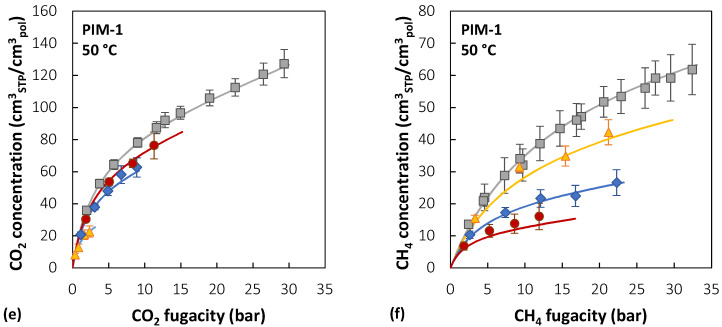
Sorption isotherms of (**a**,**c**,**e**) CO_2_ and (**b**,**d**,**f**) CH_4_ at 25, 35, and 50 °C in PIM-1 in pure- and mixed-gas conditions: grey squares, pure gas; yellow triangles, ~10% CO_2_ mixture; blue diamonds, ~30% CO_2_ mixture; red circles, ~50% CO_2_ mixture [39,40]. Solid lines are NELF model predictions [191].

Figure 7 reports the experimental sorption data of pure CO_2_, pure CH_4_, and CO_2_/CH_4_ mixtures (~10/30/50 mol% CO_2_) in PIM-1 at 25, 35, and 50 °C [39,40], together with the results of mixed-gas sorption calculations with the NELF model.

In the case of PIM-1, there is good agreement between experimental data and model predictions for CO_2_ at all temperatures and gas-phase concentrations, with average deviations below 5%, while for CH_4_, agreement increases at higher temperatures. For instance, at 25 °C, the highest relative deviation between the model and experiments is 30% in the case of an equimolar mixture, while it is reduced to 4% at 50 °C. The largest deviations for CO_2_ are always obtained in the ~10 mol% CO_2_ mixture, while for CH_4_ they are obtained in the equimolar mixture.

**Figure 8:**
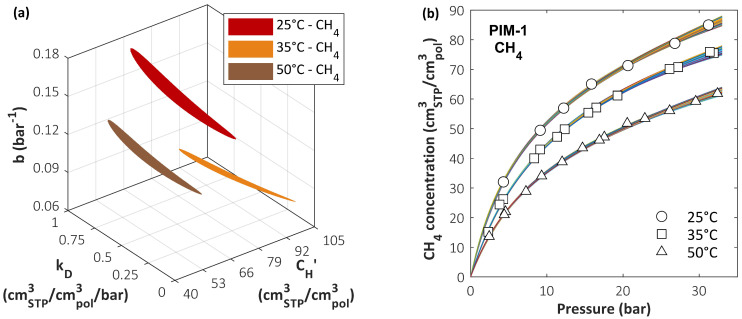
(**a**) Surfaces enclosing the range where DMS parameter sets yield a relative standard error RSE < $RSE_{max}$ in the prediction of CH_4_ sorption in PIM-1 at three different temperatures [191]; (**b**) CH_4_ sorption isotherms in PIM-1 at 25, 35, and 50 °C (experimental data from [39]) calculated with all the parameter sets enclosed by the corresponding colored regions in the plot on the left [191].

In the case of the DMS model, the same system formed by the CO_2_/CH_4_ mixture in PIM-1 has been analyzed [165]. In particular, sensitivity analysis has been carried out to analyze the error in predicting mixed-gas solubility while using two different parameter sets that have the same accuracy in predicting pure-gas sorption behavior. A comprehensive search of the parameter space was conducted using a grid method in order to identify a range of DMS model parameter values that provide equally satisfactory representations of pure-gas data. Once such a range was estimated, it was tested to determine whether different parameter sets within these confidence intervals could lead to better mixed-gas predictions than those obtained using best-fit sets. In Figure 8a, the three colored regions correspond to domains in the parameter space where the relative standard error RSE < $RSE_{max}$ (1.5%) for CH_4_ sorption in PIM-1 at three different temperatures. Each point in the colored region is a parameter set that satisfies the accuracy criterion. The bundle of calculated sorption isotherms obtained with all the parameter sets in the colored regions is reported in Figure 8b and compared to the experimental data. Although there is detectable variability in the sorption isotherms calculated using either of the parameter sets, it is always within the experimental error bars.

**Figure 9:**
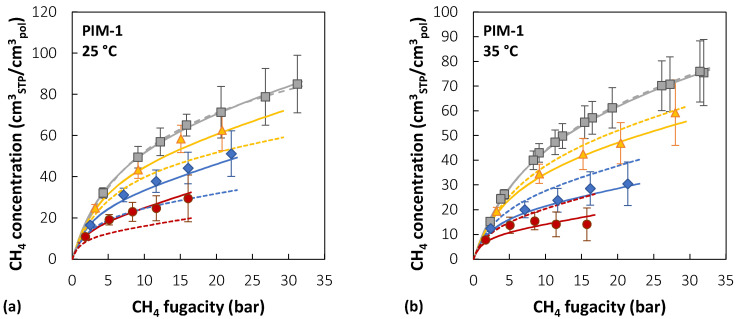

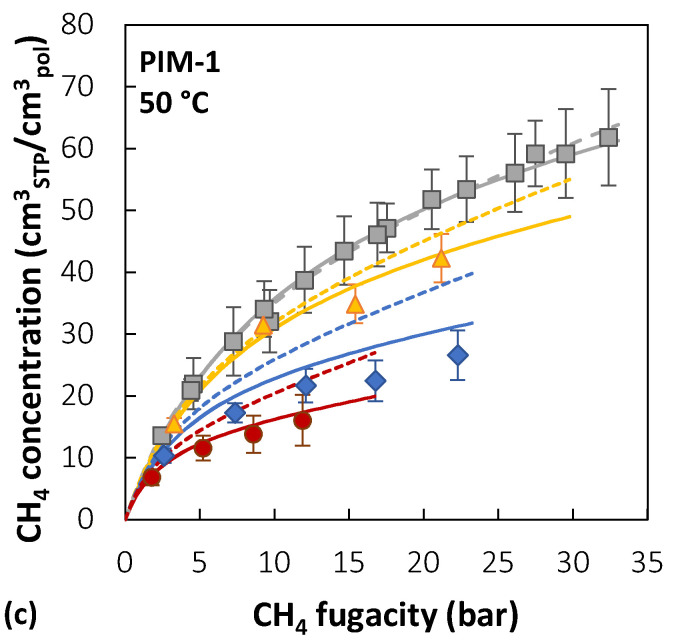
Dual-Mode Sorption model mixed-gas predictions of CH_4_ sorption in PIM-1 at (**a**) 25 °C, (**b**) 35 °C, and (**c**) 50 °C obtained with the two parameter sets reported in Table 3: solid lines correspond to Set 1; dashed lines correspond to Set 2 [191]; grey squares, pure gas; yellow triangles, ~10% CO_2_ mixture; blue diamonds, ~30% CO_2_ mixture; red circles, ~50% CO_2_ mixture [39,40].

All the parameter sets that satisfied the condition RSE < $RSE_{max}$ in the pure-gas sorption representation were used to calculate mixed-gas sorption isotherms using the best-fit values for $b_{CO_{2}}$. To quantify the accuracy of mixed-gas prediction (${\bar{RSE}}_{mix}$), the average RSE of isotherms at three concentrations (10/30/50 mol.% CO_2_) for each temperature was used, and then the lowest and the highest results were selected in order to identify the best and worst predictions, labelled, respectively, *Set 1* and *Set 2*. The parameter sets that correspond to these two extreme cases and their RSE values are summarized in Table 3. The calculated sorption isotherms are shown in Figure 9. Allowing also for experimental error, the two pure-gas representations at each temperature are deemed equivalent, and no reason for choosing one over the other can be suggested. Therefore, in the absence of mixed-gas experimental data for validation, confidence in the accuracy of the calculation is weakened. For CO_2_ sorption, the uncertainty in the mixed-gas predictions was generally lower and within the confidence region of the parameters [165]. Due to the form of the DMS model expression for the concentration, parameters $C_{H}^{\prime }$ and b are strongly coupled and, therefore, a deviation of either of them can be compensated for by a corresponding deviation of the other, yielding a similar overall quality of the fit. In order to improve the accuracy of the calculation, some authors have chosen to incorporate mixed-gas data into the fitting procedure used to retrieve the DMS parameters, obtaining different parameter sets from those retrieved considering only pure-gas data. In those cases [84,192], the representation of the mixture behavior was superior when multicomponent data were included during parametrization, but the procedure is clearly no longer predictive.

## 5. Macroscopic Models for Gas Diffusivity in Polymers

### 5.1. Free-Volume Theory

The diffusivity, or mutual diffusion coefficient, of a penetrant fluid in a polymer appears in Fick’s law to correlate the diffusive flux to the concentration gradient, representing the driving force of the phenomenon. Experimentally, diffusivity can be calculated either from transient sorption or from permeation tests. Diffusivity values vary with fluid and polymer nature and span several orders of magnitude, much more than solubility. In particular, in a fixed polymer, diffusivity decreases with vapor molecular size (molecular volume at the critical point or kinetic molecular diameter are typically considered as metrics), with slopes depending on polymer nature and microstructure, e.g., the fractional free volume. The dependence of diffusivity on penetrant size is weaker for rubbery polymers and high free-volume glassy polymers, while rigid and compact barrier polymers offer a steeper decrease of D with molecular dimensions. Diffusion is depicted as a sequence of jumps due to thermally activated movements in temporary holes in the polymer matrix. Diffusivity thus increases with free volume and temperature.

Usually, fluid diffusivity increases, often exponentially, with the concentration of fluid sorbed in the polymer due to the swelling of the matrix. Such behavior is typically encountered in rubbery or low free-volume glassy polymers. However, in glassy polymers characterized by a large excess of free volume, swelling is limited, and diffusivity may remain constant or even decrease with concentration due to saturation of the free volume [193]. In cases in which the penetrant molecules can self-associate to form clusters in the polymer, as in water-vapor diffusion in hydrophobic fluorinated ionomers such as Nafion, diffusivity can show a maximum with concentration [194]. Furthermore, sometimes diffusivity may show different values if measured from transient sorption or from permeation experiments, especially if the polymer has high free volume. Studies involving numerical simulations have attributed such phenomena to void-phase anisotropy [195].

Cohen and Turnbull [196] first showed that the self-diffusion rate of a pure fluid $D_{i,self}$ is related to the probability of finding a hole larger than the occupied volume $V^{*}$ around the molecule. Such probability is related exponentially to the average free volume $V_{F}$, which became a concept of paramount importance in the diffusion of fluids in polymers [197,198,199,200]. For glassy polymers, one has to account for excess volume due to non-equilibrium. The final expression for the self-diffusion coefficient of a fluid in a polymer is given by:

(46) $$D_{1,self}=D_{1}^{0}\exp\left(-\frac{E_{D}^{0}}{RT}\right)\exp\left\{-\gamma \frac{\omega_{1}{\hat{V}}_{1}^{*}+\xi \omega_{2}{\hat{V}}_{2}^{*}}{\omega_{1}K_{11}\left(K_{21}-T_{g1}+T\right)+\omega_{2}K_{12}\left(K_{22}-T_{g2}+T\right)}\right\}$$

where the variables and parameters involved are described in Table 4:

The theory provides expressions for the self-diffusion coefficient of a fluid in a polymer, i.e., the pure mobility. Such a value coincides with the mutual diffusion coefficient required in Fick’s law only at infinite dilution, where the sorption isotherm is linear and the activity coefficient is a constant with composition. Explanation of the meanings of the self-diffusion coefficient $D_{1,self}$, or mobility $L_{1}$, introduced in Equation (2), the mutual diffusion coefficient $D_{1}$, and thermodynamic factor $\alpha_{1}^{T}$ is reported in Appendix C. It is now sufficient to mention that, in general, the free-volume model describes the self-diffusion coefficient, and thus, the determination of mutual diffusivity requires a thermodynamic approach to estimate the thermodynamic factor, i.e., the ratio between mutual diffusion and self-diffusion:

(47) $$D_{1}=D_{1,self}\alpha_{1}^{T}$$

(48) $$\alpha_{1}^{T}=\frac{\partial \mu_{1}/RT}{\partial \ln\omega_{1}}$$

If we confine our attention to rubbery polymers, we can estimate this thermodynamic factor using the Flory–Huggins model, so that [106,107]:

(49) $$D_{1}=D_{1,self}{\left(1-\phi_{1}\right)}^{2}\left(1-2\chi_{12}\phi_{1}\right)$$

where $\phi_{1}$ is the equilibrium volume fraction of the penetrant in the polymer at the given experimental conditions. A more general correlation can be derived using other thermodynamic models, such as equation-of-state or even in the framework of the NET-GP theory, as will be shown in the following for the case of glassy polymers.

The theory thus contains a total of 10 parameters: $\left(\frac{K_{11}}{\gamma }\right)$, $\left(K_{21}-T_{g1}\right)$, $\left(\frac{K_{12}}{\gamma }\right)$, $\left(K_{22}-T_{g2}\right)$, ${\hat{V}}_{1}^{*}$, ${\hat{V}}_{2}^{*}$, $\chi_{12}$, $D^{0}$, $E_{D}^{0}$, and ξ. Independent experimental information is required to estimate these parameters:

- ${\hat{V}}_{1}^{*}$, ${\hat{V}}_{2}^{*}$ can be approximated with the molar volumes at 0 K and calculated using group contribution methods [201,202], by knowing the chemical structure of fluid and polymer, or estimated from molecular methods, such as those based on Kirkwood–Buff integrals [203,204].
- Viscosity versus temperature data for the fluid and the polymer allow calculation of *K_ij_*.
- Density data for pure fluid → $\phi_{1}$, $\phi_{2}$.
- Critical volume of pure fluid.
- Glass transition temperature of the polymer, T_g2_.

**Figure 10:**
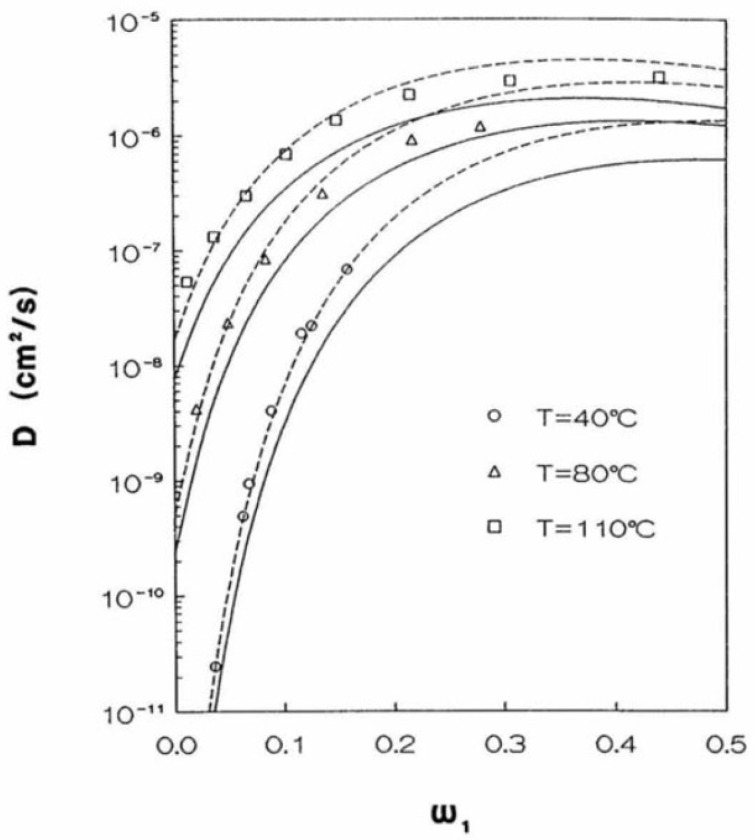
Comparison of mutual diffusion coefficients predicted from free-volume theory with experimental data for toluene/poly(vinyl acetate) systems at 40, 80, and 110 °C. Solid lines are based on the purely predictive techniques of Zielinski and Duda [206]. Dashed lines use data at 40 °C to determine *D*_0_. Reprinted with permission from Ref. [23]. Copyright 1996, CRC Press.

An example of application of the present approach to describe diffusivity in a glassy polymer can be seen in [205] and is shown in Figure 10.

The free-volume theory can be extended to multicomponent diffusion and provides expressions for the diffusion coefficient in a ternary system where the parameters involved can be estimated from volumetric, viscosity, and diffusivity data for single-component or binary systems.

The FV theory is seldom applicable as a purely predictive tool due to its high number of parameters, which are often difficult to be measured or estimated, and its application is thus limited to very few well-characterized polymer commodities (e.g., PS, PMMA, or PVC). Some useful modifications have been proposed in order to overcome such limitations. In particular, the free-volume theory was coupled to the framework of the lattice fluid model by Sanchez and Lacombe, correlating some of the parameters of the free-volume theory to the characteristic parameters of the equation of state, i.e., $T^{*}$, $p^{*}$, and $\rho^{*}$ of the penetrant molecule and the polymer matrix [207].

Furthermore, the backbone of the free-volume theory can be used effectively as a correlative tool to extend the diffusion behavior measured experimentally to wider temperature and concentration ranges. Indeed, the basic form of the present theory given by Equation (46) can be rewritten in correlative terms as follows [68,208,209,210]:

(50) $$D=A\exp\left(-\frac{B}{FFV}\right)$$

where all model parameters are replaced by adjustable parameters A and B, and the ratio between jumping unit volume and hole-free volume are expressed in terms of Fractional Free Volume, defined as:

(51) $$FFV=\frac{V_{pol}-V_{pol}^{*}}{V_{pol}}$$

which gives a measure of the free volume available for diffusion in the polymer and it is usually calculated based on group contribution methods [197]. The rationale for this simplified free-volume model for diffusivity and for the calculation of occupied polymer volume and resulting FFV has been recently analyzed and critically reviewed [211].

### 5.2. Fractal Model for Diffusion Coefficients

A fractal modelling approach, adapted from an analogous framework developed for porous media, has also been proposed to calculate gas diffusivity in polymers [185,212,213]. The diffusion coefficient D is expressed as:

(52) $$D=D_{0}\;f_{g}\;{\left(\frac{d_{h}}{d_{m}}\right)}^{2\left(D_{f}-d_{s}\right)/d_{s}}$$

where $D_{0}$ is a universal constant equal to 3.8 × 10^−7^ cm^2^/s [186,214], $f_{g}$ is the relative free volume, $d_{h}$ is the diameter of a microvoid of this volume, which can be obtained from PALS measurements, $d_{m}$ is the diameter of the penetrant gas molecule, $D_{f}$ is the general fractal dimension of the macromolecular coil representing the polymer chains and can be calculated using Equation (44), and $d_{s}$ is the polymer chain spectral dimension, which is $d_{s}$ = 1 for lineal macromolecules, while for branched/crosslinked macromolecules $d_{s}$ = 1.33 [215].

Concerning $f_{g}$, the following relation is suggested for its estimation [182,215]:

(53) $$f_{g}=0.133\left(1-\varphi_{cl}\right)$$

where the relative fraction of the closely packed segments in clusters ($\varphi_{cl}$) can be calculated using Equation (42).

This method has been applied to calculate the diffusion coefficients of several light gases and hydrocarbons both in glassy, rubbery, and semicrystalline polymers [184,185,213], achieving predictions on average within 80% [212], 50% [213], or 20% [185] of the experimental results when applied to different materials.

### 5.3. Maxwell–Stefan Model

In the Maxwell–Stefan approach to mass transfer [28], we consider the balance between the driving force acting on each species present, which is responsible for their relative motion and expressed by the chemical potential gradient, and the friction between the species (right end of the following relation). In the case of a binary mixture of components A and B:

(54) $$-\nabla \mu_{A}=\frac{RT}{Đ}x_{B}\left(v_{A}-v_{B}\right)$$

where $x_{B}$ is the mole fraction of component B, $v_{A}-v_{B}$ is the velocity of A relative to B, $\frac{RT}{Đ}$ has the physical meaning of a drag coefficient, and Đ is called Maxwell–Stefan diffusivity.

Given the definition of the molar flux $N_{i}$ with respect to a fixed reference frame:

(55) $$N_{i}={𝓃}_{t}x_{i}v_{i}$$

where $n_{t}$ is total molar concentration of the fluid mixture, Equation (54) can be rearranged as follows:

(56) $$-\frac{x_{A}}{RT}\nabla \mu_{A}=\frac{x_{B}N_{A}-x_{A}N_{B}}{n_{t}Đ}=-\left(1+x_{A}\frac{\partial \ln\gamma_{A}}{\partial x_{A}}\right)\nabla x_{A}=-\Gamma \nabla x_{A}$$

In the last two members of the equality chain, the activity coefficient $\gamma_{A}$ was introduced to express mixture non-idealities. Γ is thus a thermodynamic correction factor.

Since $J_{A}=N_{A}-x_{A}N_{A}$, with $N_{A}$ total molar flux with respect to the fixed frame of reference and considering Equation (56), we obtain:

(57) $$J_{1}=-n_{t}{Đ}_{i}\Gamma \nabla x_{A}$$

By comparing this relation to Fick’s law, one can obtain the relationship between Fick’s Diffusivity and Maxwell–Stefan Diffusivity:

(58) D=ĐΓ

When the thermodynamic correction is 1, such as in ideal mixtures, the two coincide. Đ is, in principle, independent of composition, and all composition effects are included in Γ.

In practice, a mild concentration dependence of Đ can be observed, which can be calculated with the following empirical formula from knowledge of the Maxwell–Stefan diffusivity values at the limits of the composition range:

(59) $$Đ={\left({Đ}_{x_{A}\to 1}\right)}^{x_{A}}{\left({Đ}_{x_{A}\to 0}\right)}^{1-x_{A}}$$

The mechanistic picture developed for diffusion in a two-component system can be readily extended to the general multicomponent case by considering the relative drag between all component pairs present in the mixture and a corresponding number of binary Maxwell–Stefan diffusivities:

(60) $$-\nabla \mu_{A}=RT\overset{n_{c}}{\underset{\begin{array}{c} j=1\\ j\neq i \end{array}}{\sum }}\frac{y_{j}\left(v_{A}-v_{j}\right)}{{Đ}_{Aj}}$$

In a fluid/polymer system, the permeating components are assumed to move in a fixed polymer reference system, and it is assumed that the membrane does not swell while diffusion occurs.

Recent examples of its application can be found in [141,216,217].

## 6. Explicit Models for Permeability

In the previous sections, we reviewed models able to determine gas solubility and diffusivity separately. However, these two contributions need be combined using a suitable transport scheme in the framework of solution–diffusion theory valid for homogenous amorphous polymers. This leads to the development of permeability models, which, by directly combining a description of the solubility with a formulation for the diffusion, provide explicit expressions for the penetrant flux at different temperatures, upstream pressures, or compositions.

The two main (alternative) transport schemes are Fick’s law, considering either the concentration gradient or chemical potential gradient as driving force of the process, and the Maxwell–Stefan approach. Such methods may rely on free-volume theories or other approaches to predict the value of the diffusion coefficient and require to be coupled to a suitable model for the description of solubility. Table 5 lists recent examples of the use of different transport models.

### 6.1. Partial Immobilization Dual-Mobility Model

The Partial Immobilization Dual-Mobility Model [218] applies the phenomenological description of the Dual-Mode Sorption Model (Section 4.5) to the study of gas transport. As indicated above, the model postulates the presence of two gas populations that are sorbed in Henry’s and in Langmuir’s modes with different inherent mobilities and expressed through two distinct diffusion coefficients in Fick’s law:

(61) $$N_{i}=-D_{D,i}\frac{\partial C_{D,i}}{\partial x}-D_{H,i}\frac{\partial C_{H,i}}{\partial x}$$

where $N_{i}$ is the total diffusive flux, $C_{D,i}$ and $C_{H,i}$ are the penetrant concentrations in the Henry and Langmuir regions, calculated with Equation (34), $D_{D,i}$ and $D_{H,i}$ are the diffusivities in the Henry and Langmuir regions, which are assumed to depend on temperature but not on concentration.

The expression of the permeability $P_{i}$ of a pure fluid i given by this model is:

(62) $$P_{i}=k_{D,i}D_{D,i}\left[1+\frac{F_{i}K_{i}}{\left(1+b_{i}p_{u,i}\right)}\right]$$

(63) $$F_{i}=D_{H,i}/D_{D,i}$$

(64) $$K=C_{H,i}b_{i}/k_{D,i}$$

where $k_{D,i}$ and $b_{i}$ are the Dual-Mode Sorption model parameters used in Equation (34), and $p_{u,i}$ is the upstream pressure of component i, while the downstream pressure $p_{d,i}$ is considered to be zero. To derive such expressions, local equilibrium (i.e., equality of the chemical potentials) between the Henry’s and Langmuir’s populations is assumed.

The extension to the mixed-gas case proposes expressions for the steady-state flux of each component in the mixture in terms of the various driving forces (partial pressures) and solubility and mobility coefficients. The derivation is described in detail in [218]. The final expression for the steady-state permeability of component A in a binary mixture with component B is:

(65) $$P_{A}=k_{D,A}D_{D,A}\left[1+\frac{F_{A}K_{A}}{\left(1+b_{A}p_{u,A}+b_{B}p_{u,B}\right)}\frac{p_{u,A}}{p_{u,A}-p_{d,A}}-\frac{F_{A}K_{A}}{\left(1+b_{A}p_{d,A}+b_{B}p_{d,B}\right)}\frac{p_{d,A}}{p_{u,A}-p_{d,A}}\right]$$

In the case of negligible downstream pressure, this simplifies to:

(66) $$P_{A}=k_{D,A}D_{D,A}\left[1+\frac{F_{A}K_{A}}{\left(1+b_{A}p_{u,A}+b_{B}p_{u,B}\right)}\right]$$

Symmetric expressions are obtained for component B.

If additional components are present, their effect on the permeability of component A is taken into account through expressions derived for the multicomponent mixed-gas Dual-Sorption Model.

(67) $$P_{A}=k_{D,A}D_{D,A}\left[1+\frac{F_{A}K_{A}}{\left(1+b_{A}p_{u,A}+\sum_{i\neq A}b_{i}p_{u,i}\right)}\right]$$

Recent examples of application include [219,220,221,222,223,224].

For multicomponent transport, the use of partial pressure of the various penetrants may be inadequate, especially at high pressure. Therefore, the gradient of the chemical potential of each species needs to be considered as the driving force of the process. This leads to a reformulation in the framework of the Dual-Mode model of the expression for permeability (Equation (66)) as follows [235,236], basically considering gas fugacities in place of partial pressures:

(68) $$P_{A}={k^{\prime }}_{D,A}D_{D,A}\left[1+\frac{F_{A}{K^{\prime }}_{A}}{\left(1+{b^{\prime }}_{A}f_{u,A}+\sum_{i\neq A}{b^{\prime }}_{i}f_{u,i}\right)}\right]$$

For consistency, the model sorption parameters $k_{D}$, K, and b need to be optimized considering the solubility isotherms on a fugacity basis.

### 6.2. Standard Transport Model (STM)

In 2013, a transport model was proposed by Minelli and Sarti [230,231,237,238,239] to predict permeability using the solution–diffusion framework. In particular, the model combines a simple correlation for diffusion with the NET-GP theory for sorption in polymers to derive a general framework that can be applied to predict gas permeability versus pressure for pure and mixed-gases, with the use of a few adjustable parameters. The model has predicted different trends of permeability observed experimentally, including the minimum of permeability vs. penetrant pressure that some authors have indicated as the plasticization threshold.

Let us now recall that the mutual diffusion coefficient used in Fick’s law, and thus in the solution diffusion theory, has a hybrid nature. It is convenient for modelling to decompose the penetrant diffusion coefficient $D_{i}$ as the product of a thermodynamic factor $\alpha_{i}^{T}$ and mobility coefficient $L_{i}$:

(69) $$D_{i}=\frac{\partial \mu_{i}^{NE}/RT}{\partial \ln\omega_{i}}\cdot L_{i}\equiv \alpha_{i}^{T}\cdot L_{i}$$

The NET-GP theory, e.g., the NELF model, provides the expressions to calculate the thermodynamic factor $\alpha_{i}^{T}$. The mobility $L_{i}$ may depend on penetrant concentration in the polymer. A simple exponential relation is often sufficient to describe such features, which are related to swelling and plasticization induced by the penetrant in the polymeric matrix:

(70) $$L_{i}\left(\omega_{i}\right)=L_{i,0}\cdot e^{\beta \omega_{i}}$$

The adjustable parameters $L_{i,0}$ and β, i.e., the infinite-dilution mobility coefficient and the plasticization factor, are the only ones entering the model. Their values can be determined using either permeability or diffusivity data for the fluid–polymer system.

The permeability of the penetrant i can be thus derived under the hypothesis of the upstream side of the membrane at partial pressure $p_{u,i}$ and negligible downstream pressure ($p_{d,i}\approx 0$):

(71) $$P_{i}=\frac{1}{M_{i}\;p_{u,i}}\int_{0}^{p_{u,i}}\rho_{pol}\;L_{i,0}\exp\left(\beta \omega_{i}\right)\;\frac{\omega_{i}}{p_{i}}\;Z_{i}\;dp_{i}$$

In Equation (71), $M_{i}$ is the penetrant molecular weight, $Z_{i}$ the penetrant compressibility factor (pure gas phase), and $\rho_{pol}$ the polymer density.

**Figure 11:**
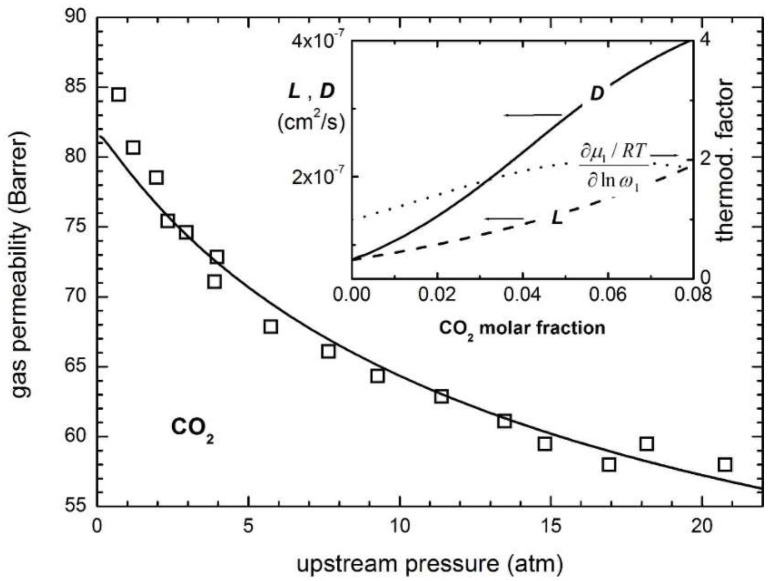
CO_2_ permeability in glassy PPO at 35 °C. Experimental data from [241]. The inset illustrates the thermodynamic factor (from NELF model), the mobility coefficient L, and the diffusivity D as functions of penetrant concentration in the polymer. Reprinted with permission from Ref. [240]. Copyright 2017, Springer.

Figure 11 shows an example of modelling CO_2_ permeability in glassy PPO [240], showing the trend of the calculated mobility and diffusivity as a function of concentration.

Such an approach has been found to be suitable to describe any type of permeation trend with upstream pressure, either decreasing or increasing and even non-monotonous behaviors [230]. The analysis of a number of different penetrants in various polymers, including high free-volume membranes, polymer commodities, or semicrystalline systems, has also allowed for the derivation of general correlations of the model parameters with the properties of pure polymers and pure penetrants [231]. It has indeed been found that mobility depends exponentially on the polymer FFV (according to a simplified free-volume theory, Equation (51)) and on penetrant molecular size (e.g., molar volume at the critical point) following an exponential law [19]:

(72) $$L_{i,0}=\frac{\tau }{V_{C,i}^{\varrho }}$$

where τ and ϱ are polymer-dependent parameters. The parameter ϱ, which describes the size-selectivity of the polymer (i.e., sieving properties), is correlated to the polymer characteristic temperature $T_{pol}^{*}$, representing the non-bonded cohesive energy of the material; exponential behavior has been found appropriate [231]:

(73) $$\varrho =\varrho_{0}\exp\left(\frac{T_{pol}^{*}}{T}\right)$$

**Figure 12:**
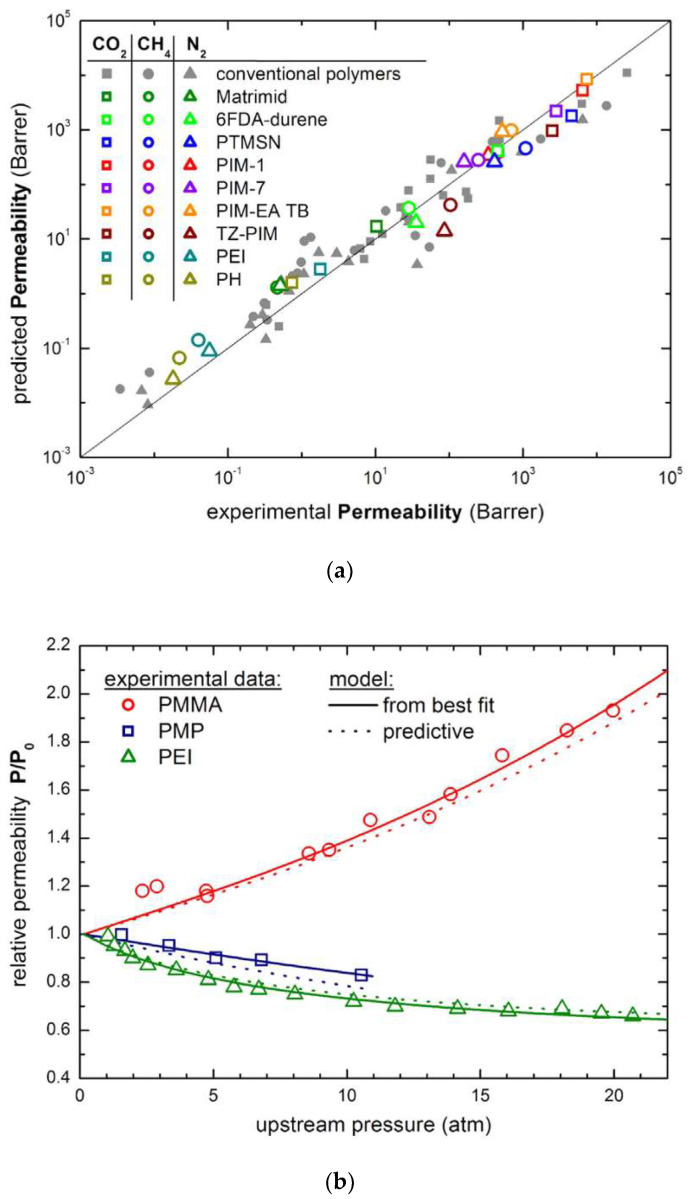
(**a**) Comparison of experimental gas permeability values at 35 °C as predicted by the model ($k_{12}=$ 0) considering CO_2_, CH_4_, and N_2_ as penetrants in various polymeric systems not considered in the development of the correlation expressed by Equation (74). (**b**) Normalized permeability of CO_2_ at various upstream pressures in glassy polymer membranes: experimental data and model calculations fitting plasticization factor in the whole curve or from a priori estimation according to Equation (74). Reproduced with permission from Ref. [231]. Copyright 2017, Elsevier.

The plasticization factor β is then closely related to the swelling induced in the polymer upon sorption, so it scales linearly with $k_{sw}$ [231]:

(74) $$\beta =K_{i}\frac{k_{sw}/S_{i}}{FFV^{2}}$$

where $K_{i}$ a proportionality constant (found to be equal to 0.85 for CO_2_), and $S_{i}$ is the solubility coefficient at infinite dilution. Therefore, the model allows the prediction, on the one hand, of the permeability at infinite dilution (Figure 12a), and its behavior with respect to upstream pressure (Figure 12b).

The description of multicomponent transport in glassy polymers by the STM approach coupled with the NELF model was reported by Toni et al. [232], who extended the model to binary mixture permeation in a stationary phase (the polymer) following a generalized Fick’s law scheme [242,243]. The diffusive fluxes for gaseous species 1 and 2 thus become:

(75) $$J_{1}=-\rho L_{1}\left(\omega_{1}\frac{\partial }{\partial \omega_{1}}\left(\frac{\mu_{1}}{RT}\right)\frac{\partial \omega_{1}}{\partial x}+\omega_{1}\frac{\partial }{\partial \omega_{2}}\left(\frac{\mu_{1}}{RT}\right)\frac{\partial \omega_{2}}{\partial x}\right)\;J_{2}=-\rho L_{2}\left(\omega_{2}\frac{\partial }{\partial \omega_{1}}\left(\frac{\mu_{2}}{RT}\right)\frac{\partial \omega_{1}}{\partial x}+\omega_{2}\frac{\partial }{\partial \omega_{2}}\left(\frac{\mu_{2}}{RT}\right)\frac{\partial \omega_{2}}{\partial x}\right)$$

in which one can recognize four different thermodynamic factors to be determined, e.g., by the EoS or NET-GP model, and the mobility coefficients of the two penetrants which are evaluated as follows:

(76) $$L_{1}=L_{1,0}\;\exp\left(\beta_{1}\;\omega_{1}+\beta_{2}\;\omega_{2}\right)\;L_{2}=L_{2,0}\;\exp\left(\beta_{1}\;\omega_{1}+\beta_{2}\;\omega_{2}\right)$$

**Figure 13:**
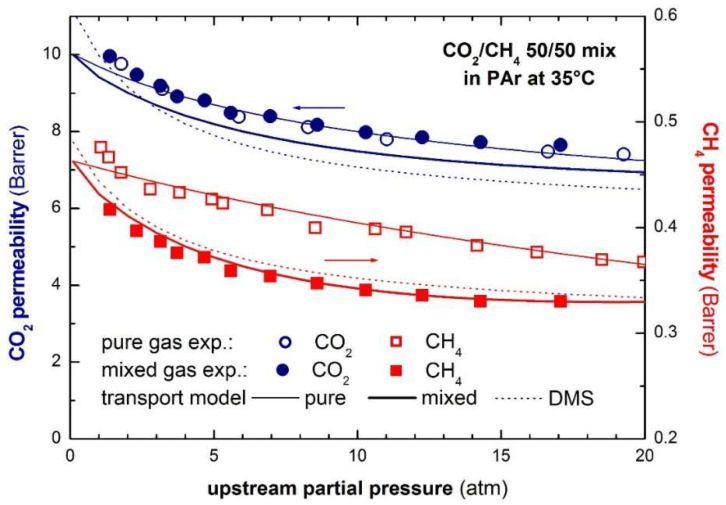
CO_2_ and CH_4_ permeability in glassy polyarylate (PAr) at 35 °C: experimental data of mixed-gas permeability by Barbari et al. [81] together with transport model curves; the corresponding experimental and model gas permeability calculated for pure CO_2_ and CH_4_ are also reported. Curves calculated using the dual-mode model are also included for comparison. Reproduced with permission from Ref. [232]. Copyright 2018, Elsevier.

The $L_{i,0}$ values at infinite dilution and the plasticization factors $\beta_{1}$ and $\beta_{2}$ were obtained from pure gas transport. Figure 13 reports the modelling of the transport of a 50/50 CO_2_/CH_4_ mixture in glassy Polyarylate using the STM approach [232].

**Figure 14:**
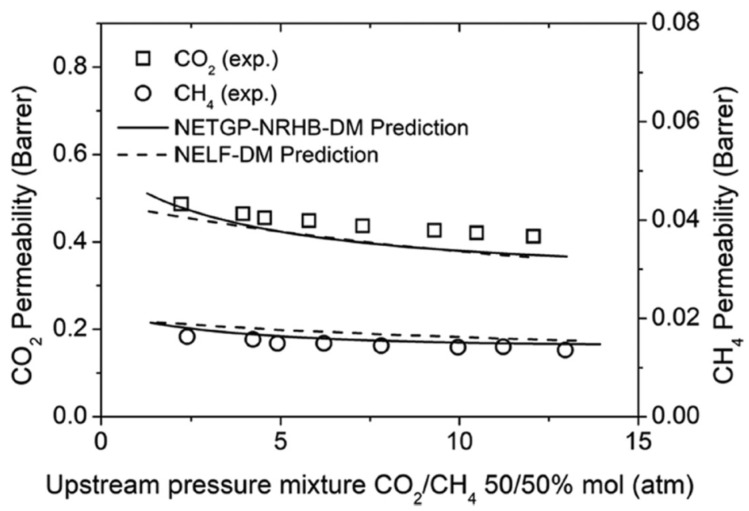
CO_2_ and CH_4_ mixed-gas permeability in polyhydroxyether (PH) at 35 °C modelling using the NE-NRHB or the NELF models within the STM approach. Reproduced with permission from Ref. [159]. Copyright 2022, American Chemical Society.

The very same transport scheme was also adopted very recently by Baldanza et al. [159], who, on the contrary, made use of a different lattice fluid equation of state (NRHB) coupled to the NET-GP theory for the thermodynamic representation of the polymer and penetrants mixture. The overall approach was thus very similar to the one discussed above, and it follows from the same assumptions, but the use of NRHB EoS may allow finer description of the polymer and penetrants, in which specific interactions may occur. As illustrated in Figure 14, only minor differences may be detected with respect to the case in which the NELF model was used.

Shoghl and coworkers [152] compared the results obtained with the standard transport model for glassy polymers with those obtained by using an expression for the diffusion coefficient derived from the dual-mode sorption relation. In the case of non/swelling agents, the two approaches yielded comparable results. For CO_2_, in some cases, the permeability predicted using NELF to calculate the thermodynamic factors in the diffusivity were significantly more accurate; in other cases, the distinction between the two approaches was not so clear. Nonetheless, these permeability predictions were obtained using a modified version of the NELF model [146], which in some cases struggles to represent the sorption isotherms of CO_2_ with the same accuracy of other gases, as reported in the study.

Samei and Raisi [233] incorporated the NELF/STM framework into a process simulation of CH_4_/N_2_ separation using five different polymers. The calculations were found to be in agreement with experimental solubility and permeability data, and the study allowed comparison of different membrane materials and process configurations, and provided recommendations in order to optimize product purity, CH_4_ recovery, total annual cost, and total capital investment, demonstrating the value of a reliable modelling framework for material properties to address practical problems.

**Figure 15:**
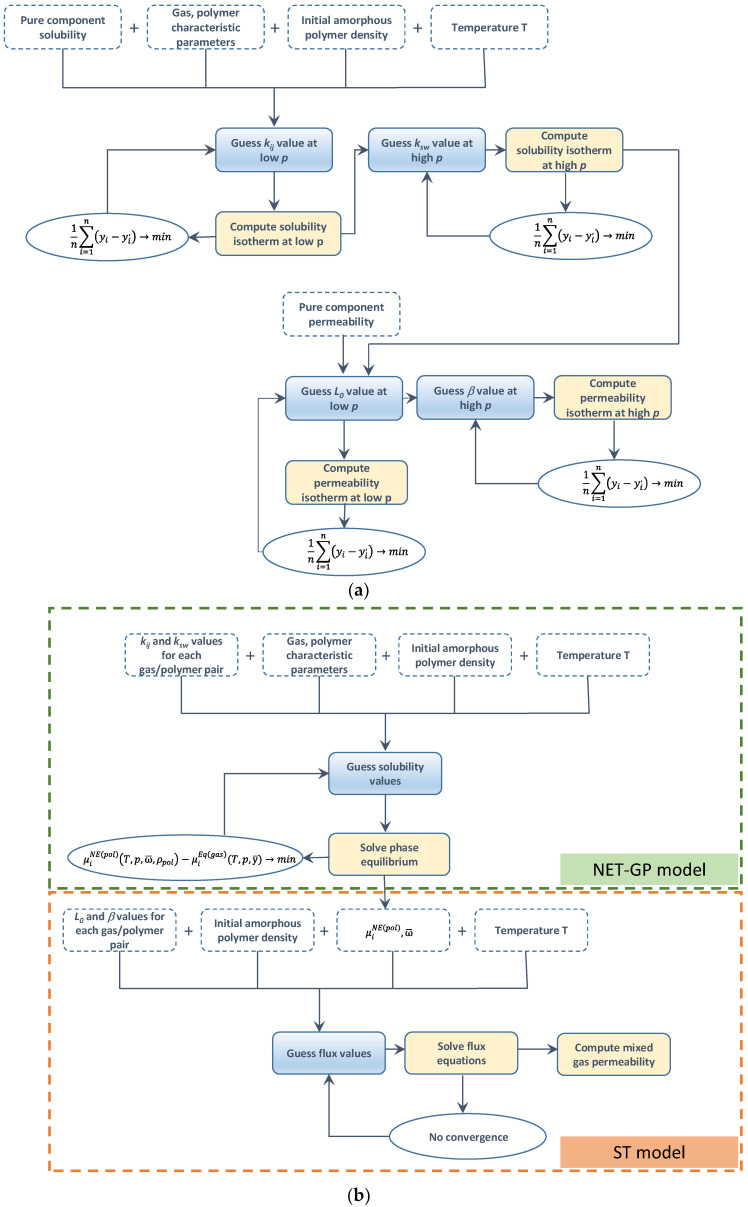
Flowchart of the data inputs (dashed boxes) and the main (colored boxes) and complementary (solid boxes) steps of the (**a**) parametrization and (**b**) mixed-gas transport prediction procedure of the ST model.

A scheme of the parametrization procedure for the STM model is reported in Figure 15.

### 6.3. Transport Models Based on the Maxwell–Stefan Approach

Krenn at al. [141] coupled the PC-SAFT EoS, combined with an elastic term to describe crosslinking, with the Maxwell–Stefan approach for diffusion in order to describe transient sorption of liquid mixtures in a highly crosslinked system. They studied the transport of water, isopropyl alcohol, and heptane through epoxy resin at different temperatures, finding good agreement with experimentally determined data, even in the case of highly anomalous sorption kinetics, such as for isopropyl alcohol.

Conversely, Ghoreyshi et al. [225] coupled the DM sorption framework with the Maxwell–Stefan approach for diffusion. They studied mixed-gas CH_4_/CO_2_ and C_3_H_6_/C_3_H_8_ transport. Their results revealed good agreement between experimental and predicted selectivities and constituted significant improvement over the Partial Immobilization Dual-Mobility model.

Marshall et al. [216] developed a model that applies DGRPT (dry glass reference perturbation theory) [147] to calculate the solubility and the thermodynamic aspects of diffusion. Pure diffusion was obtained through the Maxwell–Stefan equations, and mixture diffusion was then calculated using a simple averaging scheme on the pure fluid result. The model was applied to analyze complex liquid mixtures (e.g., alcohols, hydrocarbons, and other organic solvents) in glassy polyimide membranes for organic solvent nanofiltration applications and accurately predicted binary and complex mixture separations in glassy polymer membranes using only pure-component solubility and diffusivity as inputs.

## 7. Molecular Modelling of Gas Solubility and Diffusivity in Polymers

Since the 1980s, molecular modelling techniques have been increasingly employed to predict a wide range of properties of dense amorphous polymers, such as the thermodynamic and transport properties relevant for membrane separation [244,245,246,247]. In particular, molecular modelling studies have been instrumental in highlighting the microscopic mechanisms that are responsible for penetrant diffusion in dense rubbery and glassy polymers in terms of elementary jumps between neighboring sites of accessible volume. To calculate these properties, only models with atomistic detail are suitable because realistic representation of bonded geometry and interaction energy is required. With the development of more accurate molecular models to describe the energetic interactions of systems, new algorithms to generate amorphous polymeric structures, efficient equilibration protocols, and enhanced computational power, the reliability of predictions provided by atomistic techniques has drastically improved over the years.

### 7.1. Generation of Atomistic Models of Amorphous Polymers

In molecular models, the system is depicted as a set of particles, or interaction sites, where the action of the forces is applied and where partial point charges are located. The model may be fully atomistic (or all-atoms, (AA)), where each interaction site corresponds to an atom of the molecule; united atoms (UA), in which hydrogen atoms are considered in a single interaction site together with the atom they are bonded to; or coarse-grained (CG), in which multiple atoms are grouped together to form a single, larger interaction site.

The expression of the potential energy used to compute the forces acting on each interaction site is called a *force field*. The force field contains the contributions from bonded interactions (deviations of chemical bond lengths and bond angles as well as dihedral angles and improper torsions from their equilibrium values) and electrostatic and van der Waals interactions resulting from the interactions of electronic clouds. Parametrization of the potential energy expression is carried out from quantomechanical calculations of low-molecular-weight oligomers of the molecule (bottom-up approach), or from fitting experimental structural and/or thermodynamic properties of the material (top-down approach), [248,249,250].

Several strategies have been developed to generate dense amorphous polymer models [251,252,253,254,255]. In the recoil growth algorithm, for instance, a three-dimensional model of amorphous polymer chains is constructed to adhere to the random-coil hypothesis by Flory [256]. The initial guess configurations are generated through bond-by-bond growth of the chains under periodic boundary conditions, following the rotational isomeric state model for unperturbed chains as modified by Theodorou and Suter [257] to avoid inter- and intramolecular volume overlaps. The initial structure always undergoes molecular mechanics simulation, i.e., static minimization of the potential energy of the system, at constant volume, not considering thermal motion, to relax close contacts present in the initial guess configuration that result in unrealistically high potential energy. The minimized configurations are a good starting point for equilibration through Molecular Dynamics or Monte Carlo simulations.

An important difference also exists in molecular modelling between polymer melts and glasses: polymer melts (and rubbers) are equilibrium structures, and the probability that the system will assume a specific configuration is related to the associated potential energy V, proportional to the Boltzmann factor: $\exp\left(-V/k_{B}T\right)$. Therefore, the generation of a realistic polymer melt configuration is a well-defined problem. Polymer glasses, on the other hand, are non-equilibrium structures trapped in local energy minima dependent on their formation history. The energy barrier separating two minima are very high, and relaxation phenomena that allow the glass to transition between minimum energy configurations occur over characteristic times that are usually greater than typical simulation times. Within a minimum-energy well, the probability distribution of configurations still follows a Boltzmann distribution; therefore, the same simulation techniques for the calculation of solubility and diffusivity can, in principle, be applied both to polymer melts and polymer glasses. However, to obtain realistic results for a glass, one should average the configurations that sample different local minima of the potential energy surface. Generating microscopic configurations that incorporate the effects of formation and thermal history of a glass in a well-defined fashion, and being able to assign a probability distribution to the different minimum-energy pockets is still an open research area.

### 7.2. Molecular Dynamics (MD) Simulations

Molecular Dynamics (MD) simulations [258,259,260] consist of tracking the temporal evolution of a system through numerical integration of the equations of motion for all the interaction sites present in the system. Thermodynamic and dynamical properties of the system are computed as averages over its trajectory.

The computational cost of this technique is very high, and typical simulation times are on the order of hundreds of nanoseconds, which allows direct simulation only for processes whose dynamics are fast enough to be displayed over such a temporal interval. Even though domain decomposition techniques allow splitting of the calculations over several processors working in parallel, the aforementioned limitations persist. In this respect, challenges are posed for the equilibration of high-molecular-weight polymeric systems in general and glassy polymers in particular because their characteristic relaxation times are orders of magnitude greater than simulation times; therefore, it is necessary to start MD simulations with already-equilibrated structures.

### 7.3. Monte Carlo Simulations

In Monte Carlo (MC) simulations [258,259] a series of microscopic configurations of the system is generated conforming to the probability distribution associated with the statistical ensemble in which the simulation is carried out. At each step, starting from the previous configurations, a random perturbation is attempted. This perturbation attempt consists of an elementary move among a set of predefined possibilities, such as atom displacements, rotations, insertions, deletions of particles, and others. Monte Carlo methods can also be applied in ensembles where the number of particles can fluctuate, thus allowing the calculation of phase equilibria and, in particular, gas sorption.

The attempted perturbation is accepted or rejected according to the energy change that it entails and an acceptance criterion that ensures that the obtained sequence of microstates asymptotically samples the probability distribution of the ensemble [261] following the principle of microscopic reversibility in the generation of the Markov chain series of configurations. The properties of interest are then calculated as averages over the collection of microstates generated.

For polymeric systems, sophisticated moves have been devised that enable the overcoming of great energy barriers and allow effectively equilibration of high-molecular-weight polymeric chains of realistic experimental values, unlike MD. These moves include:

- Reptation, which consists of excising one chain end and appending it on the other side of the chain with a random torsion angle;
- Configurational bias algorithms [262,263] cut the terminal part of a chain and regrow it by avoiding the regions where volume overlaps would occur, taking this bias into account in the selection criterion;
- Concerted rotations [264] occur around seven consecutive skeletal bonds to modify change conformation without affecting bond lengths and angles;
- In addition, other connectivity-altering moves [265] have been devised, such as end-bridging [266,267], in which a trimer located in the middle of a chain is excised on one side and rotated to be attached to the end of another chain; and double-bridging [268], where two trimers are simultaneously excised from two chains and used to connect each section of the first chain with one section of the other chain.

Equilibrated polymer melt configurations obtained with this method can be used as starting points to generate glassy structures through cooling. A disadvantage of these methods is that crafting the “right” moves is a system-specific process, and they are not straightforwardly applicable to complex molecular geometries. Moreover, MC simulations do not have physical time in them, and as such, do not yield any dynamical information, such as relaxation times or diffusion constants.

### 7.4. Hierarchical Modelling Approaches

**Figure 16:**
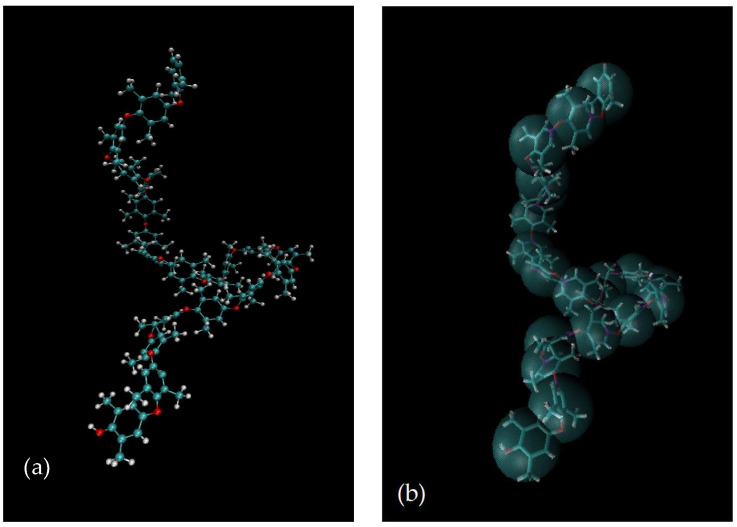
(**a**) All-atom representation of a polymer chain and (**b**) corresponding coarse-grained representation, where all atoms of the repeating unit are united into one bead.

In the case of high-molecular-weight polymers with rigid backbones and complex chemical makeup, simulations at the atomistic level of description are necessary to extract the relevant properties, but they are often inadequate to equilibrate the system to obtain a realistic configuration of the polymer. In such cases, systematic hierarchical approaches are required [269,270,271,272,273], in which the system is mapped from an atomistic to a coarse-grained level of representation by substituting a group of atoms with a single interaction site, as shown in Figure 16.

Carrying out equilibration at the coarse-grained level is more efficient for several reasons. In a CG representation, there are fewer degrees of freedom to track; therefore, longer simulations can be run with the same computational effort. The characteristic times in which the coarse-grained features change are higher compared to those at the atomistic level. For example, a coarse-grained bond length fluctuates more slowly than an atomistic bond length, which is the limiting factor dictating the choice of time-step in the integration of the equations of motion. In CG simulations, a higher time-step can be used, and therefore, longer simulation times can be achieved, allowing the system to explore a greater sample of the configuration space. A CG representation is geometrically simpler than the starting molecule; therefore, it can be equilibrated, for example, with connectivity-altering MC simulations, taking advantage of their superior effectiveness in relaxing high-molecular-weight polymers compared to MD. Nonetheless, performing CG simulations adds challenges related to the limited availability of accurate CG models for polymers and the complexity associated with deriving them. Speed and increased system size are traded off with fine-level accuracy, whose loss might be acceptable or not depending on the simulation objectives.

**Figure 17:**
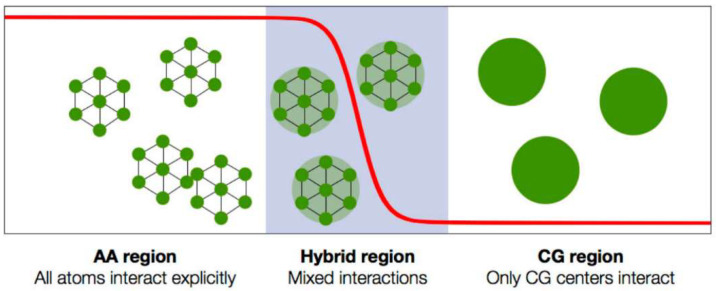
Representation of an adaptive resolution simulation in which a high-resolution fully atomistic (AA) region is coupled to a low-resolution coarse-grained (CG) region. Figure reproduced from Ref. [277] under CC-BY license terms.

Once the equilibration has been carried out, the system is back-mapped to its original atomistic representation, reconstructing the underlying geometry [274,275]. In *Adaptive Resolution* methods (represented schematically in Figure 17) [276], two spatial domains modeled at two different scales are brought together in a concurrent hybrid simulation by defining a hybrid region where particles can switch representation from coarse-grained to more-detailed and vice versa [277], depending only on a single parameter that controls the reverse mapping process and is independent of atomistic and coarse-grained force-fields.

There is not a unique way to map a system to a CG representation, and the appropriate level of coarse-graining depends on the purpose of the simulation. Recently, a *Variational Autoencoder* approach [278,279] was presented to automate the mapping choice (the Encoder section of the model) and the reconstruction of the atomistic detail (the Decoder section of the model).

In addition to a suitable structural representation, an energetic one must be derived as well. Effective CG potentials can be parameterized by reproducing macroscopic properties [280] or matching properties of the underlying microscopic representation [281,282]. Approaches used to this aim include *Iterative Boltzmann Inversion* [282], in which the fitting procedure targets the reproduction of pair correlation functions of the center-of-mass of groups of atoms corresponding to CG beads obtained from all-atom simulations. *Force matching* [283], based on fitting the potential to ab initio atomic forces of many atomic configurations, can also be used. The reverse Monte Carlo approach [284] consists of the iterative adjustment of the interaction potential to known Radial Distribution Functions that describe how the probability of finding a certain type of particle varies as a function of distance from a reference particle using a Monte Carlo simulation technique. *Relative entropy* methods [285,286] also provide a rigorous framework for multiscale simulations and offer numerical techniques for linking models at different scales. Different approaches have also been proposed, such as *hybrid particle-field* methods, using Molecular Dynamics simulations employing soft potentials derived from self-consistent field theory [287], which obtained well-relaxed all-atom polymer configurations without the need to back-map the system when changing the resolution of the representation. Ideally, CG effective potential should be transferable to thermodynamic points different from the ones used in parametrization [288,289]. In recent years, the application of machine-learning methods in force-field parametrization has yielded encouraging successes for the possibility of transferring the accuracy of first-principle methods to higher scales, thus enabling even more accurate simulations with larger sizes and time scales [290,291,292,293].

Multiscale modelling strategies can also be implemented, including continuum models. An example of coupling strategies at different resolution levels to parametrize continuum models is given by Kanellopoulos and coworkers [294], who employed molecular dynamics to calculate the Sanchez–Lacombe equation-of-state parameters of polyolefins, which they then used to perform equilibrium solubility calculations using the EoS. Another example is the *hybrid atomistic–thermodynamic scheme* to calculate gas solubility in glassy polymers [295,296]. Atomistic simulation results of the polymeric structure at conditions that are inaccessible experimentally are used to parametrize a non-equilibrium equation of state, which, in turn, is used to compute gas sorption at pure and mixed-gas conditions with negligible computational effort.

### 7.5. Simulation of Solubility

Solubility depends on the shape and distribution of free-volume elements that can accommodate the sorbing molecules; therefore, being able to provide a realistic representation of the microstructure of the polymer in terms of macroscopic density and local density, quantified, for example, through radial distribution functions, is a prerequisite. Moreover, it is necessary to have a good model to represent the energetic interactions and reproduce the relative strength in the interactions among penetrant molecules, among polymer molecules, and between penetrant and polymer molecules, and how they change as a function of penetrant concentration.

Calculation of the solubility is a phase-equilibrium calculation, with one component, the polymer, present only in one phase. There are several methods rooted in statistical mechanics principles to predict the phase equilibrium between a polymer and a multicomponent fluid mixture.

*Grand Canonical Monte Carlo* (GCMC) simulations [297] are performed at constant chemical potential, volume, and temperature: *μVT*. A bulk polymer system is simulated under periodic boundary conditions and is considered to be in contact with a gas reservoir with which it can exchange particles and energy at the specified conditions. Therefore, the number of gas particles changes during the simulation, finally fluctuating around an equilibrium value that gives the solubility. Heuchel et al. [298] proposed an application of the CGMC method to high-pressure systems by combining linearly the solubility values calculated via GCMC simulations in densely packed and pre-swollen polymer models.

The *Gibbs Ensemble Monte Carlo* method [299] can also be used to predict phase equilibria. In this method, two simulation boxes at the same temperature, each one representing one of the phases in equilibrium, are considered simultaneously. Therefore, unlike GCMC simulations, the sorbing species is modelled explicitly. Each box is built under periodic boundary conditions surrounded by replicas of itself, with no interfaces between the two systems. The total number of particles contained in both boxes and the temperature and total volume of the two boxes are kept constant, reminiscent of *NVT* simulation. Since the two boxes are at equilibrium, the algorithm ensures that the pressure and the chemical potential of each species in the two boxes, representative of the two phases, is the same. An MC simulation is performed, allowing for particle displacements, redistribution of the volume, and molecule exchanges between the boxes. During the course of the simulations, the number of atoms in each box and the volume of each box changes until reaching the values corresponding to the two coexisting phases at equilibrium. In the case of mixtures, phase equilibrium can also be simulated with MC Gibbs simulations in the *NPT* ensemble, i.e., allowing the volume of the two boxes to fluctuate but keeping the pressure constant. When dealing with dense polymer matrices or larger penetrant molecules, the acceptance probability of inserting a molecule is drastically lower, and techniques relying on these moves yield unreliable results.

Monte Carlo simulations in the *Semigrand Canonical Ensemble* can also be performed to calculate phase equilibria [300]. In these simulations, identity-exchange trial moves among the various species are considered, i.e., moves in which two particles of different species are not displaced, but their identity is swapped. This method is effective in equilibrating the concentration of solutions for which the various species have comparable sizes; therefore, it is not suitable for gas-polymer systems.

*Hybrid Monte Carlo* simulations [301] include performing a short MD trajectory as one of the possible moves. This is advantageous to sample local conformational changes, especially in the case of complex systems where traditional MC moves have low acceptance probability. Hybrid Monte Carlo simulations performed in the osmotic ensemble (*N_pol_f_gas_PT*) [302,303,304,305,306] also account for swelling effects [307]. However, at high pressure, where pronounced dilation is typically observed, a *sequence of separate CGMC and MD simulations* is often preferred to iteratively relax the polymer density [308]. The discussion of the simulation of gas-induced swelling is expanded in Section 7.7.

In *Grand Canonical Molecular Dynamics* [309,310], phase equilibrium is computed from two different simulations performed for the pure condensed phase (*NPT*) and for a system in which the number of polymer chains is kept fixed but fractional penetrant gas molecules are exchanged between the system and a reservoir. A fractional molecule is a molecule whose potential energy of interaction with the rest of the system is scaled by a coupling parameter, λ, ranging between zero and one. The solution of the appropriate equations of motion for this ensemble [310] that governs the exchange of molecules between the system and the material reservoir yields the final value of the coupling parameter as 0 or 1, meaning deletion or full insertion of the gas particle into the system. *Continuous Fractional Component Monte Carlo* follows a similar strategy, improving the efficiency of the rescaling of λ with the introduction of an adaptive bias potential [311].

One widely used technique to obtain infinite dilution solubility coefficients is the *Widom test particle insertion* method [312]. A “ghost” penetrant particle is positioned in the polymer matrix at random positions and orientations, and its interaction energy with the other particles present in the system is computed. From that, the excess chemical potential of the penetrant inside the polymer, $\mu_{i}^{ex}$, can be determined. In turn, from the expression for $\mu_{i}^{ex}$, one can obtain the solubility coefficient straightforwardly, according to Equation (78):

(77) $$\exp\left(-\frac{\mu_{i}^{ex}}{RT}\right)=\frac{\left\langle V\exp\left\{-1/k_{B}T\left[\Delta U_{\mathrm{test}}^{\mathrm{inter}}\right]\right\}\right\rangle }{\left\langle V\right\rangle }$$

(78) $$\frac{1}{S_{i}}=\frac{\rho RT}{M_{i}}\underset{x_{i}\to 0}{\lim}\left[\exp\left(-\frac{\mu_{i}^{\mathrm{ex}}}{RT}\right)\right]\;$$

where $k_{B}$ is the Boltzmann constant, T is the temperature, and $\Delta U_{\mathrm{test}}^{\mathrm{inter}}$ is the change in the intermolecular energy of the system brought by the insertion of the additional molecule (i.e., the potential energy of interaction between the test molecule and the other molecules of the system). The polymer is not allowed to relax its configuration as a consequence of the insertions. This method can be applied by post-processing a sequence of microstates originating in the course of an MD or MC simulation, by performing several ghost insertions in each configuration. *Excluded-volume map sampling* [313,314] and *grid search* methods [315] can be implemented for dense systems in order to increase the sampling efficiency by avoiding inserting particles in densely packed regions. However, as the system becomes denser and the solute molecules become bigger, the probability of a successful insertion without overlap with existing molecules drops dramatically, and, therefore, the estimate of the solubility through Widom insertions becomes less reliable.

Strategies proposed to mitigate this issue include the use of *configurationally biased* [316] *bond-by-bond* insertions of the penetrant molecules, or the use of *particle deletion* moves instead of particle insertions (Staged Particle Deletion [317], Direct Particle Deletion [318]).

Alternatively, the *free-energy perturbation* method can be applied, where a coupling parameter is introduced between the solute–matrix interactions, and solubility is obtained by thermodynamic integration [319] over a series of simulations conducted at different values of the coupling parameter. The *expanded ensemble* scheme [320] can be implemented to calculate free-energy differences between thermodynamic states and can be considered as an application of the free-energy perturbation method but within a single simulation.

Another technique is *extended ensemble MD* [321], where the coupling of the solute with the rest of the system dynamically changes, allowing the solute to escape from low-energy pockets and sample the phase space more efficiently. A minimum-to-minimum mapping method [322] takes into account local configurational changes to accommodate an inserted molecule to lower superpositions and excluded volume effects. Another approach suitable for sorption of large molecules in dense matrices is the *fast-growth thermodynamic integration* method [323], which allows efficient determination of chemical potential from several independent thermodynamic integration runs.

**Figure 18:**
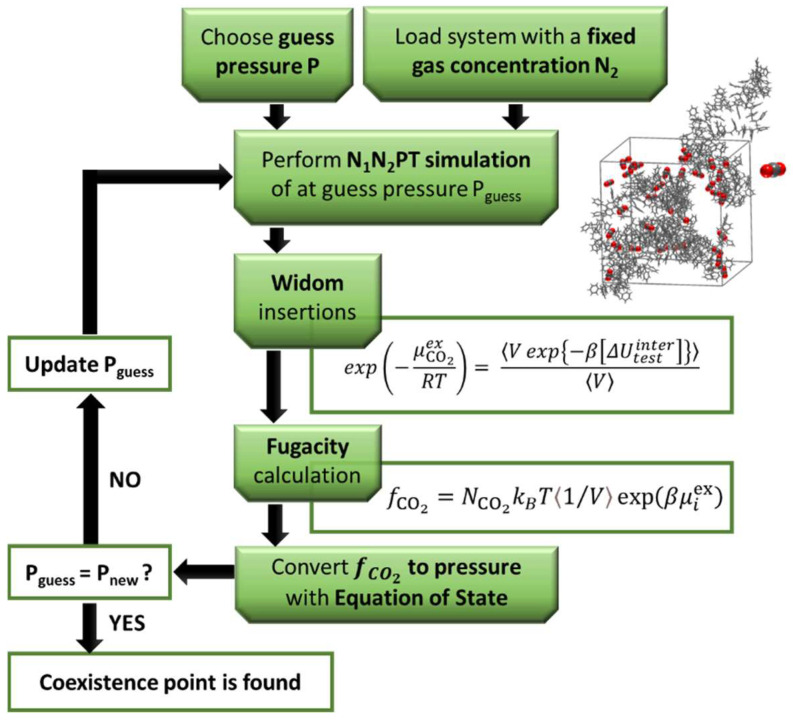
Schematic representation of the iterative *NPT*/Widom algorithm for molecular simulation of gas solubility in polymers at high pressure.

Sorption isotherms up to high pressure can be calculated through a series of Monte Carlo simulations performed in the osmotic ensemble [302,303,304,305,306] (constant number of polymer particles, temperature, and pressure, and constant fugacity of the penetrant *f_gas_*, which can be preemptively calculated with an equation of state). This method allows direct simulation of polymer swelling, since volume changes are admissible moves. This technique also has the advantage of not explicitly simulating the gas phase. Alternatively, an iterative scheme can be implemented [119,324], performing *NPT* MD simulations of the polymer–penetrant system with a fixed number of polymer chains *N_pol_* and gas molecules *N_gas_*, i.e., at fixed composition and at a guessed pressure value. After the *NPT* MD simulation, the trajectory is post-processed to evaluate the excess chemical potential, for example by performing Widom insertions. The excess chemical potential is then related to penetrant fugacity and to the pressure of the system. The obtained value is used to carry out a new *NPT* MD simulation at the same composition and at the new pressure. The procedure is repeated until a coexistence point with consistent pressure and composition is obtained, as schematically displayed in Figure 18.

### 7.6. Simulation of Diffusivity

**Figure 19:**
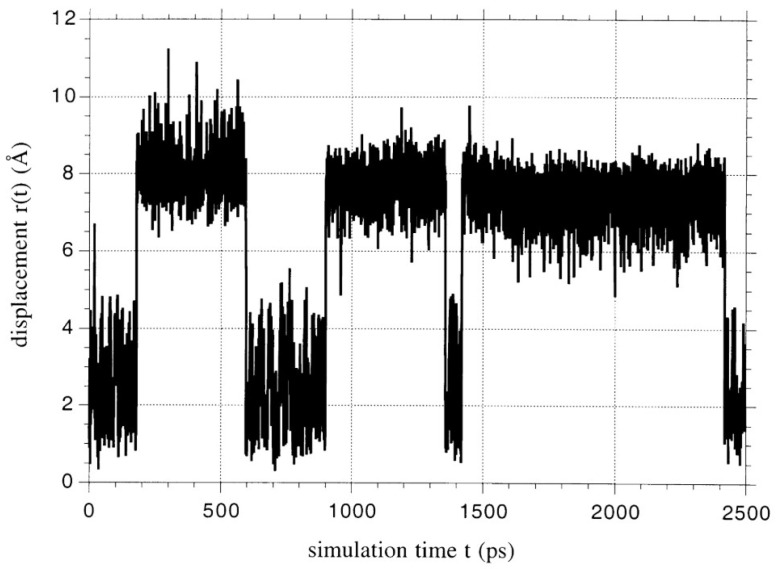
Displacement of an O_2_ molecule in a glassy polyimide during an MD simulation: the molecule jumps back and forth between two adjacent cavities and fluctuates within a cavity in-between jumps. Reprinted with permission from Ref. [329]. Copyright 2000, Elsevier.

Molecular simulations have provided useful insight into the mechanism of diffusion in polymeric materials. Computer simulations have revealed that the transition from a pore-flow regime, typical of microporous membrane materials, to a molecular diffusion regime, typical of dense systems, occurs at pore dimensions of 5–10 Å, comparable to polymer chain spacing [325]. Below this value, permeation is no longer a pressure-driven flow through tiny pores, but a diffusive process controlled by the motion of the polymer chains. Two distinct mechanisms have been identified [325]. In the case of melts [326,327], the thermal motion of the polymer chains randomly opens and closes “gaps” capable of accommodating the penetrant molecules very rapidly, and a dissolved penetrant molecule will be displaced into these neighboring cavities, diffusing with a characteristic time dictated by the frequency of density fluctuations of the order of the penetrant size. On the other hand, in the glassy state, the cavities are more permanent over time [328], and a molecule will be trapped moving back and forth into a void until an opening of sufficient size is created by thermal fluctuations of the polymer (Figure 19). Therefore, for a glass, the diffusivity depends on the distribution of cavities located at a distance that can be travelled by the penetrant when a connecting path is opened and on the frequency of this event. 

Accurately capturing both structure and mobility of the polymer is thus necessary to obtain reliable diffusion constants. Actually, this infrequent jump process is not confined to diffusion in polymers below $T_{g}$. It has also been shown in melts and rubbery polymers when the temperature is sufficiently low for the distribution of accessible volume regions to remain relatively unchanged over the time scale of a penetrant jump [325,327,330].

**Figure 20:**
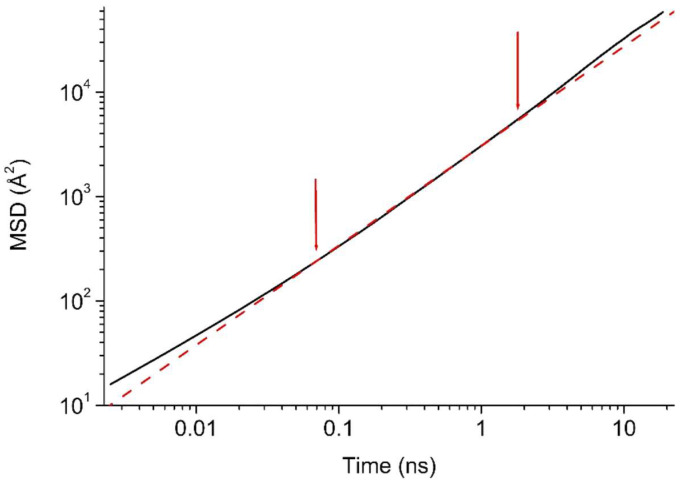
Black: example of MSD vs. time for gas diffusion in a molten polymer. Red: the dashed line with slope = 1 and the two arrows mark the region of Fickian diffusion.

Self-diffusivity $D_{i,self}$ measures the displacement of a molecule as a result of random thermal motion, and it is proportional to the mean squared displacement (MSD) of the molecule [331], averaged over all molecules.

(79) $$D_{i,self}=\underset{t\to \infty }{\lim}\frac{\left\langle (r_{i}\left(t\right)-r_{i}\left(0\right){)}^{2}\right\rangle }{6t}$$

where $\left(r_{i}\left(t\right)-r_{i}\left(0\right)\right)$ is the distance travelled by a molecule from the initial time to time t. Self-diffusivity corresponds to the penetrant mobility defined in Equation (2). Binary diffusivity coincides with self-diffusivity in the limit of low concentration (infinite-dilution regime). It represents the same physical property defined in the formulation of the free-volume theory in Section 5.1. Operatively, at finite time conditions, the calculation of self-diffusivity through Equation (79) requires identification of the portion of the simulation for which diffusion follows the Fickian or normal regime [23] (slope of the MSD equal to 1 in a logarithmic plot of MSD vs. time, as exemplified in Figure 20). The slope of the best-fit line to the Fickian portion of the MSD curve divided by 6 (in 3-dimensional space) yields the value of the diffusion coefficient.

Under the assumptions that the velocity correlations between different molecules are negligible and the self-diffusivity of the polymer is much smaller than that of the penetrant, one obtains the relation between self-diffusivity, or mobility, and mutual diffusivity $D_{i}$ [303] reported in Equation (4).

**Figure 21:**
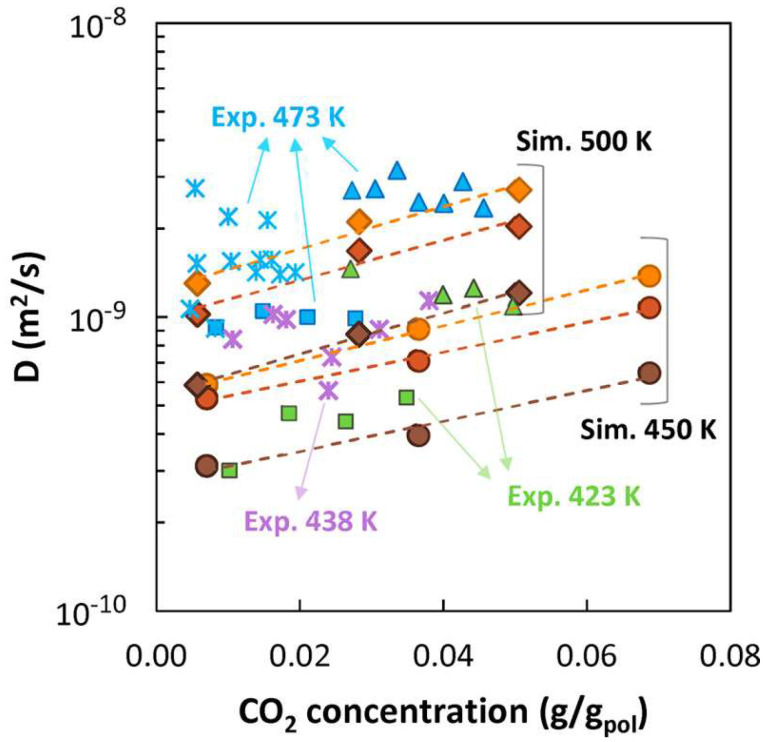
Simulated CO_2_ diffusion coefficients in atactic polystyrene as a function of concentration at different temperatures and *M*_w_: circles, 450 K; diamonds, 500 K; orange, *M*_w_ of 2100 g/mol; red, 5200 g/mol; brown, 31,000 g/mol. Comparison with experimental data (squares [332], triangles [333], stars [334]; blue 473 K, green 423 K, purple 438 K). Figure reproduced from Ref. [119] under CC-BY license terms.

For small-molecular-weight penetrants in polymer melts, diffusivities are usually high enough that they can be captured within the timespan accessible to an MD simulation by tracking the mean squared displacement of the penetrant molecules (Figure 21).

At realistic application conditions, the diffusion of gases occurs with the presence of a concentration gradient within the polymer. With *Non-Equilibrium Molecular Dynamics* (NEMD) [258,335], an external driving force is imposed on the system, so that it is kept out-of-equilibrium and the penetrants move inside the matrix under the action of the driving force. For small external forces, the system remains in the linear response regime, and the transport properties at steady state can be computed from the ratio of the flux to the acting driving force. In the case of gas transport in polymers, Müller-Plathe et al. [336] compared MD and NEMD results for diffusion of He, H_2_, and O_2_ in amorphous poly(isobutylene), but did not detect a substantial computational gain. On the other hand, NEMD was found more efficient than equilibrium MD to obtain the diffusivity of penetrants in liquids and microporous sorbents [337].

When the temperature of the system is below the glass-transition temperature of the polymer, gas diffusivity very often becomes too slow to be predicted by MD. Indeed, this is a consequence of the mechanism of diffusion in these conditions, since the motion of the gas that is being tracked consists mostly of rattling back and forth inside a specific free-volume microvoid, whereas displacement into a neighboring void, which truly contributes to diffusion, occurs very rarely. Therefore, it is impossible to obtain statistically significant information about these jumps by performing brute-force MD simulations. In these cases, *Transition State Theory* of infrequent events (TST) can be adopted [338,339,340]. Implementing TST to calculate penetrant diffusivity in a polymer matrix involves the identification of transition states between free-volume elements and how that system can evolve from one state to another. In the potential energy surface of the system, the transition state is a saddle-point that can be crossed, moving from one state to another, both identified as local minima separated by a high-energy barrier. TST enables the calculation of rate constants for the transition between states based on the probability of the system to be in the transition state between two states compared to the probability of being in its initial state. This method was first applied by Gusev and Suter [341] in the case of a rigid polymer and subsequently extended to also account for elastic vibrations of polymer atoms [342]. It was further generalized by Greenfield and Theodorou [343] by the inclusion of polymer degrees of freedom into calculation of transition states and diffusion pathways, therefore taking into account the local chain motions that accompany the formation of a passage between neighboring free-volume elements. Further extensions of the method allow handling of complex shapes and chemical constitutions both for the penetrant and the polymer [344]. Once the network of possible states and connecting pathways and the rate constants of transitions between states have been determined, the diffusivity can be obtained considering a Poisson process of successive uncorrelated penetrant jumps between states. A Kinetic Monte Carlo (KMC) simulation can be performed to solve the master equation representing the time evolution of the probability that the system is in a particular state [345].

Additionally, for simulated diffusion coefficients, it is important to be mindful of finite-size effects [346]. Indeed, it has been shown that the diffusion coefficients computed with MD simulations scale linearly with the inverse of the simulation box length Λ [347], and the following analytic finite-size correction was developed, which goes by the name of *Yeh–Hummer* correction [348]:

(80) $$D_{i,self}^{\infty }=D_{i,self}^{MD}+\frac{k_{B}T\Xi }{6\pi \eta \Lambda }$$

where $D_{i,self}^{MD}$ is the finite self-diffusion coefficient computed in the MD simulations, $k_{B}$ is the Boltzmann constant, T is the absolute temperature, η is the shear viscosity computed in MD simulations, and Ξ is a dimensionless constant equal to 2.837298 for periodic (cubic) lattices [349]. In addition to LJ fluids and water, the validity and applicability of the Yeh–Hummer correction has been demonstrated for a variety of systems, including Lennard-Jones fluids, water, carbon dioxide, n-alkanes, and deep eutectic solvents [346]. Recently, Jamali et al. also derived a generalized form for finite-size corrections of diffusion for multicomponent mixtures [350]. Each system is affected by finite-sized effects differently, and the magnitude of the effect further depends on the thermodynamic point [351,352]. Unless short polymer chains are considered, or conditions that significantly decrease system viscosity, such as very high temperatures or very high gas concentrations, the Yeh–Hummer correction is expected to be negligible for the diffusivity of gases in polymers. Indeed, Moultos et al. [352] observed that the magnitude of the correction decreased at increasing molecular weight for short oligomers.

### 7.7. Gas-Induced Swelling

The dilation induced by the gases upon sorption inside polymer matrices influences both the thermodynamic and the transport properties of the system; therefore, it is important to account for such effects during the simulation of materials, especially in far from infinite dilution conditions. Some of the methodologies presented to simulate solubility allow the direct inclusion of these effects in the calculation, e.g., Hybrid Monte Carlo simulations performed in the osmotic ensemble [303], Continuous Fractional Component Monte Carlo simulations performed in the osmotic ensemble [353], iterative *NPT* MD–Widom insertion steps [119,324], and iterative *NPT* MD–GCMC steps [308]. These methods can be applied to obtain equilibrated gas–polymer systems also suitable for the simulation of gas diffusion at high pressure. In the case of the linear combination of results from dense and pre-swollen systems proposed by Heuchel et al. [298], while for solubility, the agreement with experimental data was satisfactory, for diffusivity, it was only qualitative. Indeed, an accurate representation of the structural properties of the material, thus a direct, rather than indirect, inclusion of swelling effects in the simulation, seems to be a necessity for the accurate prediction of gas diffusivity [119].

### 7.8. Atomistic Simulation of Gas-Separation Membranes

In recent years, atomistic molecular modelling techniques have proven very useful to investigate the structure and dynamics of dense, amorphous membrane polymers and transport processes in these materials [244,245,246,354]. Gas transport in rubbery and glassy polymers has been studied with a variety of approaches, both for pure- and mixed-gas conditions.

The first simulation studies of gas sorption and transport in polymers were performed on materials with a simpler chemical makeup than those showing competitive gas separation performance nowadays; however, they served as benchmarks for the development of methods and algorithms that were subsequently also applied to innovative polymeric materials of interest for these applications.

#### 7.8.1. Bulk Systems Simulations

**Figure 22:**
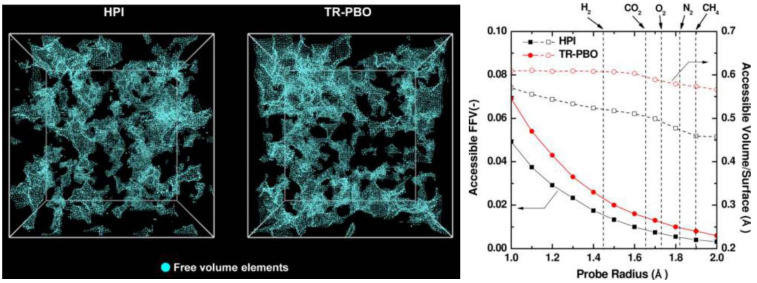
Simulated free-volume distribution of a low-FFV polyimide (**left**) and its high-FFV thermally rearranged variant (**center**). (**Right**) Fractional free volume (FFV) and ratio of free-volume and surface area with probe radius calculated from kinetic diameters of gas molecules. Reproduced with permission from Ref. [361]. Copyright 2014, American Chemical Society.

Detailed molecular analyses of the solubility and diffusivity of small gases has been reported in the literature for glassy polyamides [355], poly(amide imide)s [355,356,357] and polyimides [355,357,358,359,360,361], polysulfones [298,362], polyurethanes [363] high free-volume polyacetylenes [364,365], and rubbery materials [355,357,366,367,368], often using the GCMC method to evaluate solubility and the TST method or the analysis of MSD of the gas molecules to evaluate diffusivity, with good agreement with experimental measurements. Thermally Rearranged (TR) polybenzoxazoles are among the best-performing materials for gas separation, and for this class of polymers also, the use of molecular simulations has provided useful insight, in particular regarding the free-volume size distribution and topology following the thermal rearrangement process, and how this is correlated to the enhanced permeability shown by these materials [361,369,370,371] (Figure 22). Indeed, analysis of the size distribution of free-volume elements and comparison with measurements of positron annihilation lifetime spectroscopy (PALS) allowed the rationalization of correlations between polymer chemistry, microstructure, and gas transport properties. For materials whose permeability is dominated by diffusion rather than sorption effects, molecular simulations have shown the effect of pore-size distribution for materials with similar fractional free volume [372]. Moreover, important differences in the diffusion mechanisms of rubbery and glassy polymers concerning the lifetime of channels between free-volume elements allowing for molecular jumps and the average residence time of gas molecules in each free-volume element have been highlighted [355].

Molecular modelling has also been employed to obtain insight on the molecular origin of the structural features of amorphous polymers measured by wide-angle x-ray scattering [373,374] and d-spacing [375], as well as to establish structure–property correlations for more-rational material design [346,376,377,378].

**Figure 23:**
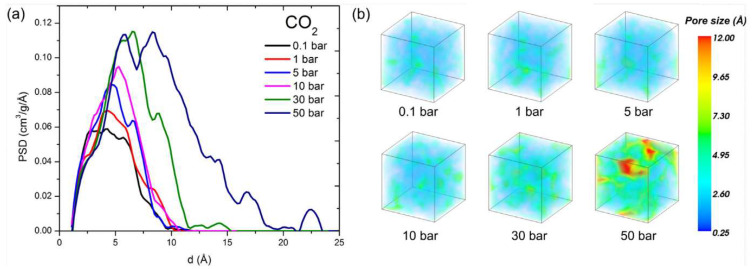
Simulated (**a**) pore-size distribution of PIM-1 and (**b**) 3D pore-size visualization as a function of CO_2_ uptake from molecular simulations. Reproduced with permission from [382]. Copyright 2018, Elsevier.

Heuchel et al. were the first to apply TST to the study of gas transport in PIM-1, simulating He, H_2_, Ar, O_2_, N_2_, CH_4_, CO_2_, and Xe sorption and diffusion [379]. Solubility tended to be overestimated in the simulations by a factor 2 to 3 (5 for CO_2_), with the exception of He and H_2_, for which good agreement with experimental data was found. Simulated diffusivities were overestimated by a factor 2 for the light gases, while they were closer to experimental values for the other gases, with the exception of CO_2_, which was one order of magnitude lower than the experimental value. This is ascribed to the fact that the spherical representation used for all gas molecules is unrealistic in the case of CO_2_, which is a markedly linear molecule. Different methods and molecular representations have obtained more-accurate results for this system. For example, Fang et al. [380,381] applied the Widom Insertion method to predict CO_2_ solubility in PIM-1, obtaining close agreement with experimental data. Recently, Kupgan et al. [382] employed a scheme combining Grand Canonical Monte Carlo and Molecular Dynamics simulations devised by Hölck et al. [308] to simulate CO_2_ sorption in PIM-1 up to 50 bar and analyze its effect on the pore-size distribution of the polymer (Figure 23).

Lanchet et al. [307] used hybrid MC simulations in the osmotic ensemble to study the sorption of CH_4_, CO_2_, H_2_S, H_2_, N_2_, O_2_, and H_2_O in PVDF at infinite dilution at 493 K. No experimental data were available for validation, but trends were consistent with those in other works. Chen et al. combined ab initio calculation, Molecular Dynamics, and Monte Carlo simulations to investigate the structural characteristics and transport behavior of CO_2_, CH_4_, O_2_, and N_2_ in PIM-Trip-TB and KAUST-PI-1 membranes [383], showing the capability of atomistic techniques to also correctly represent the properties of rigid polymeric structures of complex chemical constitution [384,385]. Heuchel et al. analyzed glassy polysulfone and poly(ether sulfone) with CO_2_ gas pressures up to 50 bar at 308 K [298]. Pre-swollen packing systems were prepared based on experimental dilation data, and sorption was determined using GCMC. Sorption isotherms with satisfactory accuracy were determined by combining the solubilities obtained for swollen and unswollen systems. Gas diffusivity was also determined using TST; in this case, the results obtained for the preswollen systems were only qualitatively consistent. A similar strategy was employed to study swelling during nitrogen adsorption isotherms at 77 K for five PIM variants [386] by pre-swelling the simulation boxes up to 15%, finding that the size of the free-volume elements increased with the simulated swelling percentage, while the closely packed polymer chains remained tightly associated. Neyertz et al. [387] performed extensive molecular dynamics simulations of several fluorinated polyimides with CO_2_ weight percentages up to 30%. Diffusion coefficients were estimated from a trajectory-extending kinetic Monte Carlo method. Diffusivity values and activation energies were found to be in good agreement with experimental data. Swelling effects, together with hysteresis effects related to sorption–desorption cycles, which affected CO_2_ diffusivity as well, were quantified during the simulation.

In order to obtain plasticization-resistant membranes, crosslinking is often employed to tighten the material and to prevent significant swelling upon sorption. Strategies to build molecular models of highly crosslinked polymer networks have been developed [388,389,390,391,392,393,394] and validated against measurements of apparent Brunauer–Emmet–Teller (BET) specific surface areas, crosslinking degrees, porosity, and sorption measurements. Moreover, simulations have shown the evolution of porosity throughout the crosslinking process [388] and the formation process of membranes prepared through interfacial polymerization [395].

#### 7.8.2. Gas–Polymer Interface Simulations

**Figure 24:**
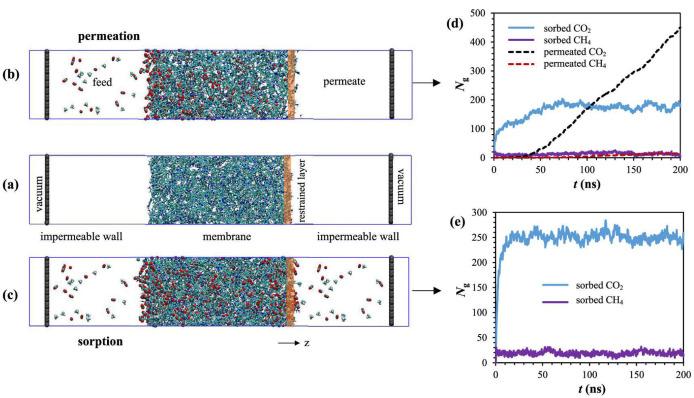
Simulation of CO_2_/CH_4_ mixture permeation through a thin membrane layer. (**a**) Membrane with a thin layer position-restrained along the *z*-axis; (**b**) system for permeation of the CO_2_/CH_4_ mixture; (**c**) system for sorption of the CO_2_/CH_4_ mixture; (**d**) number of sorbed and permeated gas molecules during permeation; (**e**) number of sorbed gas molecules during sorption. Reproduced with permission from Ref. [399]. Copyright 2019, American Chemical Society.

Interface simulations are less common that bulk system simulations, but are nonetheless of great importance because they yield unique insight into the molecular interfacial mechanisms of sorption and transport. Anderson et al. [396] employed NEMD to examine transport of methane and *n*-butane molecules in the bulk and interface regions of polyethylene, poly(4-methyl-2-pentyne), and polydimethylsiloxane (PDMS), developing correlations to calculate penetrant diffusivity and permeability from the accessible cavity fraction and average amplitude of chain oscillations of the polymers. Frentrup et al. [397] performed NEMD simulations for the direct simulation of He and CO_2_ permeability through a thin membrane of PIM-1 with good qualitative agreement with experimental data (Figure 24). Additional interface studies [398,399] are discussed in the next section.

#### 7.8.3. Mixed-Gas Simulations

**Figure 25:**
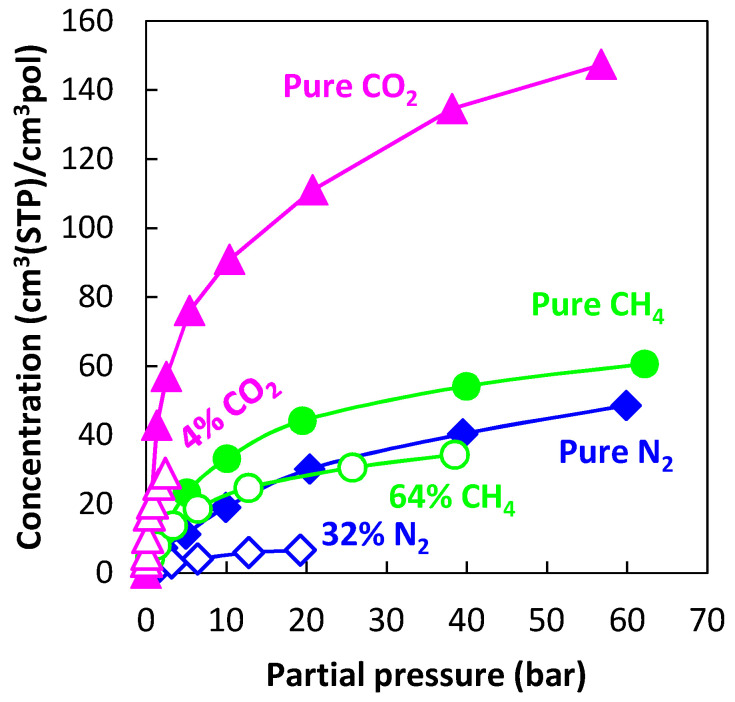
Simulated pure- and mixed-gas sorption (4:32:64 CO_2_/N_2_/CH_4_) in 6FDA-6FpDA. Results from Neyertz and Brown [403].

Fewer modelling studies have analyzed mixed-gas sorption effects. Recently, Rizzuto et al. [400] coupled GCMC atomistic simulations and Ideal Adsorbed Solution Theory (IAST) [401] to investigate the mixed-gas permeation properties of CO_2_/N_2_ mixtures in thermally rearranged polymers. Pure-gas sorption of both gases was underestimated by the simulations. However, competitive effects between the components in the mixture, expected in the case of glassy polymers, were displayed and found to greatly affect the solubility of the less-condensable gas of the mixture. Neyertz and Brown [398] performed large-scale MD simulations of air separation with an ultra-thin polyimide membrane surrounded by an explicit gas reservoir. In this work, they determined gas solubility, diffusivity, and O_2_/N_2_ selectivity at multicomponent conditions, comparing favorably with experimental results. Multicomponent solubility-selectivity was found to be comparable to the ideal one. Tanis et al. [402] studied CH_4_/N_2_ separation with several polyimide membranes using atomistic simulations. Solubility coefficients obtained from excluded-volume map sampling test-particle insertions were combined with diffusion coefficients calculated with a variant of the kinetic Monte Carlo approach. Iterative procedures allowed accounting for swelling effects upon sorption, both in pure- and mixed-gas cases. Their results highlighted non-ideal behavior in the multicomponent case, affecting both the predicted permeability and selectivity of the membrane material. Furthermore, competitive sorption effects in CH_4_/N_2_ and CH_4_/N_2_/CO_2_ in a polyimide were simulated using three different iterative techniques [403], which were compared in terms of accuracy and computational efficiency. Competitive sorption effects can be observed in the simulation results (Figure 25), and the authors obtained correlations between exclusion effects and sorbed concentration that are consistent with experimental evidence [41].

Liu et al. [399] investigated the separation performance of a thin membrane of a branched PIM-1 architecture for CO_2_/CH_4_ mixtures. They performed a large-scale direct simulation of permeability incorporating both polymer flexibility and membrane plasticization during gas permeation. Hart et al. studied a hypothetical functionalized polymer of intrinsic microporosity with an ionic backbone (carboxylate) and extra-framework counterions (Na^+^) for CO_2_ gas storage and separation applications [404]. They evaluated CO_2_/CH_4_ and CO_2_/N_2_ mixed-gas separation performance with GCMC simulations, finding very appealing performance under several industrially relevant conditions.

Molecular modelling is mature enough to go hand-in-hand with the experimentation process to synthesize new materials, making it more informed and rational [378,405,406]. A wealth of detailed and reliable information about the microscopic characteristics and macroscopic behavior of a system can be extracted by the implementation of molecular simulation strategies. Finally, one of the most appealing applications of molecular modelling is the preliminary large scale screening of different molecular structures for a specific application even before the hypothetical structures are synthesized, and several studies of this kind have been performed on a smaller scale for polymeric gas separation materials [376,377,407,408,409,410]. Predictive simulation of the change in structural features associated with variation in the chemical constitution and calculation of the corresponding gas transport properties highlights structural property correlations and provides guidelines for the future design of new chemical structures with the desired properties.

## 8. Conclusions

Membrane technology can enable sustainability in an expanding array of processes, and thus it is at the forefront of many environmental challenges that are being addressed worldwide. Efficient membrane materials design is a necessary prerequisite for the development of better-performing green-alternative separations. In this regard, material modelling and simulations play a key role in enabling disruptive innovation and helping to compact the lab-to-market cycle as much as possible.

In this work, macroscopic and molecular modelling approaches for the study of gas sorption and transport in polymeric membranes have been reviewed.

The solution–diffusion mechanism allows membrane performance (permeability and selectivity) to be calculated from its solubility and diffusivity contributions, which can be obtained separately, with uncorrelated methods.

This was the prevailing approach in the field of macroscopic modeling of membranes in the past, and many sophisticated models were proposed, the most notable being the NET-GP approach for solubility and the free-volume theory for diffusion. Such a methodology began being replaced in the last decade with more comprehensive approaches in which permeability is estimated explicitly by considering a suitable combination of models or correlations for solubility and diffusivity components. A good example of this new conceptual development is the Standard Transport Model. Macroscopic methods are computationally efficient, although robust algorithms are needed when multicomponent mixtures are considered and when complex models such as the SAFT Equation of State for solubility are used. The accuracy of such methods in predicting membrane permeability and selectivity is satisfactory with a relatively small number of binary parameters, e.g., two for the simulation of light-gas permeability in rubbery membranes and four for the simulation of swelling gases, such as CO_2_ in a glassy membrane. No additional binary parameters are required for multicomponent mixtures.

On the other hand, since its early days, molecular modelling has been a powerful tool for phenomenological analysis, and has been instrumental in elucidating the bulk transport mechanisms of small molecules in both rubbery and glassy polymers and in establishing structure–property relationships in a fully predictive fashion. The quantitative accuracy of molecular simulations continually increases as more sophisticated simulation algorithms are introduced, better force fields are developed, and higher available computational power enables the simulation of larger systems and longer time spans.

Most approaches focus, as above, on separate simulations of fluid solubility and diffusivity in the bulk amorphous structure. However, direct simulations of permeation and simulations of interface phenomena are also increasingly of interest, to shed light on additional features related to membrane separation that cannot be easily probed with other techniques. Nevertheless, atomistic simulation of membrane separation performance is still hindered by the high computational effort required to equilibrate solid polymeric phases, especially glassy ones and molecular weights closer to the experimental conditions. Therefore, the development of more-generalized multiscale or hierarchical molecular modelling paradigms that leverage coarse-grained representations is of paramount importance to streamline the simulation of more-realistic polymeric systems and to fulfill the ambition of a computationally driven membrane materials discovery pipeline.

With the increasing popularization of data-driven methods in the natural sciences and engineering domains, it can be expected that this third branch of computational analysis will receive more investigation efforts and will complement and intertwine with existing empirical and physics-based methods to provide valuable new insights to the membrane research community, and will assist in the rational design of tailored materials for old as well as new membrane separations.

## Figures and Tables

**Table 1:** Overview of the most-employed models for fluid solubility in polymers. The definition of all variables and symbols can be found in Appendix B.

Material	Model Type	Common Version	System Representation	What It Provides		Pure Component Parameters *	Adjustable Binary Parameters ^#^	Extension to Multicomponent?
**Rubbery polymer**	Activity coefficient model	Flory Huggins (FH)	Lattice	$G^{ex}$	Solubility isotherm	1 per component (liquid density)	$1:\;\chi_{ij}$	Yes
	Equation-of-State	Lattice Fluid (LF) [86,104]	Compressible Lattice 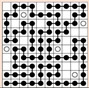	G; Equation-of-State	Solubility isotherm; Swelling isotherm	3 per component $\left(p^{*},T^{*},\rho^{*}\right)$	$1:\;k_{ij}$	Yes
		Non-random Hydrogen Bonding (NRHB) [87,88]				3 per component $\left(p^{*},T^{*},\rho^{*}\right)$ + 2 for each association interaction ($E_{\alpha \beta }^{0}$ $,\;S_{\alpha \beta }^{0}$)		
		Statistical Associating Fluid Theory (SAFT) [105]	Hard sphere chains 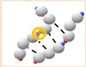	A; Equation-of-State	Solubility isotherm; Swelling isotherm	3 per component (σ,ε,M/n + 2) for each association interaction $(\varepsilon_{AB}$$,\;k_{AB}$)	$1:\;k_{ij}$	Yes
**Glassy polymer**	NET-GP [102]	NE-LF, NE-SAFT, NE-PC (SAFT), NE-NRHB	The same of the corresponding equilibrium EoS model ^†^	$G^{NE}$	Solubility isotherm	Same as EoS model used + 1 (polymer density) ^‡^	$1\;(k_{ij}$$)\;+\;1\;(k_{sw}$) ^§^	Yes
	DMS		Two populations of sorbed molecules in Henry and Langmuir modes		Solubility isotherm	-	$3:\;k_{D},C_{H}^{\prime },b$	Yes
	Fractal				Solubility	4 for polymer ($T_{g},\;K,\;C_{S},A_{cr}$), 2 for fluid $\left(\frac{\varepsilon }{k_{B}},F_{g}^{ef}\right)$	$1:\;S_{0}$	Yes, but it requires multicomponent data
**All polymers**	GAB				Solubility isotherm	-	$3:\;v_{m},h,p^{*}$	Yes, but it requires multicomponent data

* Obtained from pure component data, e.g., LV equilibrium for fluids or pressure–volume–temperature data for polymers; ^#^ Obtained from binary data, e.g., solubility or heat of mixing of the fluid in the polymer for fluid/polymer couples, LV equilibrium data for fluid–fluid pairs at a specific temperature. ^†^ For example, the underlying representation for the non-equilibrium system will be “Compressible Lattice” if the corresponding equilibrium model chosen is the LF one, or “Hard Sphere Chains” if one chooses an equilibrium model from the SAFT family, etc. ^‡^ For example, the pure component parameters of the NE-LF model will be: $p^{*},T^{*},\rho^{*}$, $\rho_{pol}$; ^§^ $k_{sw}$ is only required for fluid–polymer pairs, and it is 0 when swelling is negligible.

**Table 2:** Multicomponent effects documented during mixed-gas sorption in polymeric membranes. Polymer acronyms are defined in Appendix A.

Polymer	Mixture (More-Soluble Component First)	Predominant Multicomponent Effect Observed at Fixed Fugacity	Ref.
**PDMS (rubbery)**	CO_2_/CH_4_	Swelling	[45]
	n-C_4_H_10_/CH_4_	Swelling	[47]
**XLPEO (rubbery)**	CO_2_/C_2_H_6_	Swelling	[48]
**PMMA**	CO_2_/C_2_H_4_	Competition	[49,50,51]
	CO_2_/N_2_O	Competition	
**PPO**	CO_2_/CH_4_	Competition	[46]
**CTA**	CO_2_/CH_4_	Competition	[52]
**PTMSP**	CO_2_/CH_4_	Competition	[53]
	n-C_4_H_10_/CH_4_	Competition	[54]
**6FDA-TADPO**	CO_2_/CH_4_	Competition	[27]
**6FDA-mPDA**	CO_2_/CH_4_	Competition	[38]
**HAB-6FDA**	CO_2_/CH_4_	Competition	[32]
**TR450**	CO_2_/CH_4_	Competition	[32]
**PIM-1**	CO_2_/CH_4_	Competition	[39,40]
	C_2_H_6_/CH_4_	Competition	[41]
	C_2_H_6_/CO_2_	Competition	[41]
	C_2_H_6_/CO_2_/CH_4_	Competition	[41]
**TZ-PIM**	CO_2_/CH_4_	Competition	[42]
**PIM-Trip-TB**	CO_2_/CH_4_	Competition	[43]

**Table 3:** DMS model fugacity-based parameter sets used to calculate mixed-gas sorption of CO_2_ and CH_4_ in PIM-1 reported in Figure 9.

	T (°C)	$k_{D,CH_{4}}$ $\left(\frac{cm_{STP}^{3}}{cm_{pol}^{3}bar}\right)$	$C_{H,CH_{4}}^{\prime }$ $\left(\frac{cm_{STP}^{3}}{cm_{pol}^{3}}\right)$	$b_{CH_{4}}$ $\left({\mathrm{bar}}^{-1}\right)$	*RSE_pure_*	${\bar{RSE}}_{\mathrm{mix}}$
Set 1	25	0.940	66.52	0.174	1.5%	13%
	35	0.084	100.6	0.078	1.5%	7%
	50	0.317	67.15	0.094	1.5%	13%
Set 2	25	0.428	89.57	0.115	1.5%	27%
	35	0.622	73.15	0.108	1.5%	23%
	50	0.763	47.05	0.140	1.5%	21%

**Table 4:** Variables and parameters in the self-diffusion coefficient expression given by the Free-Volume Theory (Equation (46)).

$D_{1,self}$	Self-diffusion coefficient of fluid (1) in polymer (2)
$D_{1}^{0}$	Pre-exponential factor = diffusion in a fluid with infinite free volume
$E_{D}^{0}$	Energy required for a jump into an adjacent free volume void
γ	Coefficient accounting for overlap of free volume available to adjacent molecules (0.5–1).
$V_{i}^{*}$	Occupied volume
$V_{F}$	Average free volume per jumping unit
$\xi \equiv \frac{V_{1}^{*}}{V_{2}^{*}}$	Ratio between occupied volumes of the fluid (1) and the polymer (2)
$K_{1i},K_{2i}$	Parameters related to pure component viscosity for component *i*

**Table 5:** Sample of recent gas-transport modelling studies, divided by model category.

		Model for Diffusion		
		Fick’s law ∇c	Fick’s law ∇μ	Maxwell–Stefan
**Model for Sorption**	Dual-Mode Model	**Partial Immobilization Model** [218] (Section 6.1) Moon at al. 2020 [219] Park et al. 2020 [220] Balçık et al. 2021 [221] Miandoab et al. 2021 [222] Suhaimi et al. 2021 [223] Lim et al. 2022 [224]	Shoghl et al. 2021 [152]	Ghoreyshi et al. 2015 [225] Monsalve-Bravo et al. 2019 [226] Mathias et al. 2021 [227]
	Thermodynamic Model (LF, SAFT, FH…)	LF/NELF—Shoghl et al. 2017 [228] FH—Bounaceur et al. 2017 [229]	**Standard Transport Model** [230] (Section 6.2) NRHB—Baldanza et al. 2022 [159] NELF—Minelli et al. 2017 [231] NELF—Toni et al. 2018 [232] NELF—Shoghl et al. 2021 [152] NELF—Samei et al. 2022 [233] PC-SAFT—Liu et al. 2020 [137]	FH—Krishna et al. 2016 [234] PC-SAFT—Krenn et al. 2020 [141] FH—Mathias et al. 2021 [227] PC-SAFT—Marshall et al. 2022 [216]

## Data Availability

Not applicable.

## Appendix A. Acronyms List

PDMS	Poly(dimethyl siloxane)
XLPEO	Crosslinked Poly(ethylene oxide)
PMMA	Poly (methyl methacrylate)
PVDF	Polyvinylidene fluoride
PPO	Poly(2,6-dimethyl-1,4-phenylene oxide)
CTA	Cellulose triacetate
PTMSP	Poly(1-trimethylsilyl-1-propyne)
6FDA-TADPO	Hexafluoro dianhydride–3,3,4,4-tetraaminodiphenyl oxide polypyrrolone
6FDA-mPDA	4,4′-(hexafluoro isopropylidene) diphtalic dianhydride-m-phenylenediamine
HAB-6FDA	3,3′-dihydroxy-4,4′-diamino-biphenyl 2,2′-bis-(3,4-dicarboxyphenyl) hexafluoropropane dianhydride
TR450	Thermally rearranged derivative of HAB-6FDA
PIM-1	Polybenzodioxane
TZ-PIM	Tetrazole-modified PIM-1
PIM-Trip-TB	Ladder polymer of intrinsic microporosity including a triptycene group
RSE	Relative Standard Error
EoS	Equation of State
LF	Lattice Fluid
NET-GP	Non-Equilibrium Thermodynamics for Glassy Polymers
NELF	Non-Equilibrium Lattice Fluid
SAFT	Statistical Associating Fluid Theory
NRHB	Non-random Hydrogen Bonding
DMS	Dual-Mode Sorption
GAB	Guggenheim−Anderson−de Boer
STM	Standard Transport Model
LJ	Lennard-Jones
BET	Brunauer–Emmet–Teller
MD	Molecular Dynamics
NEMD	Non-Equilibrium Molecular Dynamics
IAST	Ideal Adsorbed Solution Theory
MC	Monte Carlo
GCMC	Grand Canonical Monte Carlo

## Appendix B. Other Symbols and Variables

***Generalities***	
$J_{i}$	Molar flux of component i (gaseous species diffusing across the solid membrane)
$P_{i}$	Permeability (coefficient) of component i (gaseous species diffusing across the solid membrane)
$p_{i}$	Partial pressure of component i in the gas mixture
$f_{i}$	Fugacity of component i in the gas mixture
l	Membrane thickness
$c_{i}$	Penetrant molar concentration in the membrane phase
$c_{u,i}$ $,\;c_{d,i}$	Molar concentration (moles of gas/membrane volume) in the membrane phase on the upstream and downstream sides, respectively
$D_{i}$	Local diffusivity or diffusion coefficient of species i in the membrane
*z*	Coordinate indicating the position along the membrane thickness
$L_{i}$	Mobility (or self-diffusion diffusivity) of species i in the membrane
$\mu_{i}$	Chemical potential of species i
$S_{i}$	Solubility coefficient of species i in the membrane $S_{i}=\frac{∆c_{i}}{∆p_{i}}$
${\bar{D}}_{i}$	Concentration-averaged diffusivity of species i in the membrane
$\omega_{i}$	Mass fraction of the fluid in the membrane
$\alpha_{i,j}$	Selectivity of component i versus component j in the membrane
$\alpha_{i,j}^{S}$	Solubility-Selectivity of component i versus component j in the membrane
$\alpha_{i,j}^{D}$	Diffusivity-Selectivity of component i versus component j in the membrane
$S_{0}$	Pre-exponential factor for solubility coefficient
$\Delta H_{s}$	Heat of sorption
$D_{0}$	Pre-exponential factor for diffusion coefficient
$E_{D}$	Activation energy of diffusion
$P_{0}$	Pre-exponential factor for permeability coefficient
$E_{P}$	Activation energy of permeation
$\Delta H_{c}$	Heat of condensation of the fluid species
$\Delta H_{m}$	Heat of mixing of the fluid species in the membrane
***Robeson’s upper bound***	
$\beta_{i,j}$	Gas couple-dependent parameter (position of the upper bound)
$\lambda_{i,j}$	Gas couple-dependent parameter (slope of the upper bound)
$d_{k,j}$	Kinetic diameter of the larger molecule
$d_{k,i}$	Kinetic diameter of the smaller molecule
a	Parameter used in the correlation for $\beta_{i,j}$ and $\lambda_{i,j}$
𝓫	Parameter used in the correlation for $\beta_{i,j}$ and $\lambda_{i,j}$
𝓯	Parameter used in the correlation for $\beta_{i,j}$ and $\lambda_{i,j}$
***Activity coefficient models for solubility***	
$\gamma_{i}$	Activity coefficient (of the fluid in the membrane)
$n_{i}$	Number of moles of component i in a mixture
$G^{ex}$	Excess Gibbs free energy of a mixture
${\bar{G}}_{i}^{ex}$	Partial molar excess Gibbs free energy for a component i in a mixture
***LF EoS***	
G	Gibbs free energy
N	Total number of molecules
$k_{B}$	Boltzmann’s constant
$n_{c}$	Number of components (gases + polymer)
$M_{i}$	Molar mass of component i
$\rho_{i}$	Density of component i
$v_{i}^{*}$	Molar volume of a lattice cell of component i
$r_{i}^{0}$	Number of lattice cells occupied by a molecule of pure component i
$\varepsilon_{i}$	Non-bonded interaction energy between two lattice cells occupied by component i
$T_{i}^{*}$	Characteristic temperature of component i $T_{i}^{*}=\frac{\varepsilon_{i}}{k_{b}}$
$p_{i}^{*}$	Characteristic pressure of component i $p_{i}^{*}=\frac{\varepsilon_{i}}{v_{i}^{*}}$
$\rho_{i}^{*}$	Characteristic density of component i $\rho_{i}^{*}=\frac{M_{i}}{r_{i}v_{i}^{*}}$
${\tilde{T}}_{i}$	Reduced temperature of component i ${\tilde{T}}_{i}=\frac{T}{T_{i}^{*}}$
${\tilde{p}}_{i}$	Reduced pressure of component i ${\tilde{p}}_{i}=\frac{p}{p_{i}^{*}}$
${\tilde{\rho }}_{i}$	Reduced density of component i ${\tilde{\rho }}_{i}=\frac{\rho_{i}}{\rho_{i}^{*}}$
ρ	Density of the mixture
$\tilde{T}$	Reduced temperature $\tilde{T}=T/T^{*}$
$\tilde{p}$	Reduced pressure $\tilde{p}=p/p^{*}$
$\tilde{\rho }$	Reduced density $\tilde{\rho }=\rho /\rho^{*}$
$k_{ij}$	Binary interaction parameter between i and j
$\omega_{i}$	Mass fraction of component i
$\phi_{i}$	Volume fraction of component i in close-packed conditions $\phi_{i}=\frac{\omega_{i}/\rho_{i}^{*}}{\sum_{i}^{N}\omega_{i}/\rho_{i}^{*}}$
$\rho^{*}$	Characteristic density of the mixture $\frac{1}{\rho^{*}}=\overset{n_{c}}{\underset{i}{\sum }}\frac{\omega_{i}}{\rho_{i}^{*}}$
$p^{*}$	Characteristic pressure of the mixture $p^{*}=\overset{n_{c}}{\underset{i}{\sum }}\phi_{i}p_{i}^{*}-\overset{n_{c}-1}{\underset{i}{\sum }}\overset{n_{c}}{\underset{j>i}{\sum }}\phi_{i}\phi_{j}\Delta p_{ij}^{*}$ $∆p_{ij}^{*}=p_{i}^{*}+p_{j}^{*}-2\left(1-k_{ij}\right)\sqrt{p_{i}^{*}\cdot p_{j}^{*}}$
$T^{*}$	Characteristic temperature of the mixture $T^{*}=\frac{p^{*}}{\sum_{i}^{N}\frac{p_{i}^{*}\phi_{i}}{T_{i}^{*}}}$
$v^{*}$	Average close-packed molar volume in the mixture $v^{*}=\frac{T^{*}R}{p^{*}}$
$r_{i}$	Number of lattice cells occupied by a molecule in mixture $r_{i}=\frac{r_{i}^{0}v_{i}^{*}}{v^{*}}$
***NRHB EoS***	
$\varepsilon_{i}^{*}$	Characteristic energy in the LF model and in the NRHB model
$\varepsilon_{i,h}^{*}$	Enthalpic contribution to the characteristic energy
$\varepsilon_{i,s}^{*}$	Entropic contribution to the characteristic energy
$E_{\alpha \beta }^{0}$	Association energy between group α and a functional group β
$S_{\alpha \beta }^{0}$	Association entropy between a functional group α and a functional group β
***SAFT EoS***	
$A^{res}$	Residual Helmholtz free energy (at fixed temperature and volume)
$A^{hs}$	Hard-sphere term of residual Helmholtz free energy
$A^{disp}$	Dispersion term of residual Helmholtz free energy
$A^{chain}$	Chain term of residual Helmholtz free energy
$A^{assoc}$	Association term of residual Helmholtz free energy
$\mu_{i}^{IG}$	Chemical potential of species *i* in the ideal gas state
***NET-GP model for solubility***	
$\mu_{i}^{NE}$	Non-equilibrium chemical potential of species i
$\mu_{i}^{Eq}$	Equilibrium chemical potential of species i
*ρ_pol_*	Non-equilibrium density of the glassy polymer
$\mu_{i}^{NE\left(pol\right)}$	Non-equilibrium chemical potential of species i in the polymer phase
$\mu_{i}^{Eq\left(gas\right)}$	Equilibrium chemical potential of species i in the gas phase
$\bar{\omega }$	Composition vector in the polymer phase
$\bar{y}$	Composition vector in the gas phase
$\rho_{pol}^{0}$	Non-equilibrium density of the dry glassy polymer
$k_{sw,i}$	Swelling coefficient correlating the glassy polymer density to the gas i partial pressure
***DMS model for solubility***	
$k_{D,i}$	Henry’s law constant
$C_{H,i}^{\prime }$	Langmuir capacity constant
$b_{i}$	Langmuir affinity constant
$k_{D0}$	Pre-exponential factor for temperature-dependence of $k_{D}$
$b_{0}$	Pre-exponential factor for temperature-dependence of b
$∆H_{D}$	Enthalpy of sorption for Henry’s mode of sorption
$∆H_{b}$	Enthalpy of sorption for Langmuir’s mode of sorption
***GAB model for solubility***	
v	Penetrant adsorbed mass ratio
p	Adsorbate pressure
$p^{*}$	Reference pressure value associated with the adsorbate
$v_{m}$	Capacity of the 1st adsorption monolayer
h	Dimensionless binding parameter
$r_{ij}$	Parameter related to sorbate–sorbate interactions in multicomponent GAB
$h_{ij}$	Parameter related to sorbate–sorbate interactions in multicomponent GAB
***Fractal model for solubility coefficient***	
$F_{g}^{ef}$	Effective cross-sectional area of the sorbed gas molecules
$D_{f}$	Global fractal dimension parameter
$S_{0}$	Minimum solubility of a gas molecule
$\varphi_{cl}$	Relative fraction of the closely packed segments in clusters
$T_{g}$	Polymer glass transition temperature
$X_{cr}$	Degree of crystallinity
$d_{f}$	Fractal dimension of the polymer structure
$A_{cr}$	Cross-sectional area of a macromolecule
$C_{S}$	Characteristic ratio representing the index of chain flexibility
***Free-volume theory for diffusion coefficient***	
$D_{1,self}$	Self-diffusion coefficient of fluid 1 in polymer 2
$D_{1}^{0}$	Pre-exponential factor = diffusion in a fluid with infinite free volume
$E_{D}^{0}$	Energy required for a jump into an adjacent free-volume void
γ	Coefficient accounting for overlaps of free volume available to adjacent molecules
$V_{i}^{*}$	Occupied volume
$V_{F}$	Average free volume per jumping unit
$\xi \equiv \frac{V_{1}^{*}}{V_{2}^{*}}$	Ratio of occupied volumes
$K_{1i},K_{2i}$	Parameters for component i related to pure component viscosity
$\alpha_{1}$	Thermodynamic factor of mutual diffusivity
FFV	Fractional free volume
A,B	Adjustable parameters for correlation between diffusivity and FFV
$V_{pol}$	Polymer-specific volume
$V_{pol}^{*}$	Polymer-occupied volume
***Fractal model for diffusion coefficient***	
$D_{0}$	Universal constant of the model, equal to 3.8 × 10^−7^ cm^2^/s
$f_{g}$	Relative free volume
$d_{h}$	Diameter of a microvoid
$d_{m}$	Diameter of the penetrant gas molecule
$d_{s}$	Polymer chain spectral dimension
***Maxwell–Stefan model for diffusion coefficient***	
Đ	Maxwell–Stefan diffusivity
$v_{A}-v_{B}$	Velocity of species A relative to species B
Γ	Thermodynamic correction factor
$N_{i}$	Total molar flux of species i (with respect to a fixed reference frame)
${𝓃}_{t}$	Total molar concentration of the fluid mixture
$x_{i}$	Mole fraction of component i in the mixture
***Partial Immobilization Dual-Mobility Model for Permeability***	
$N_{i}$	Total molar flux of component i
$C_{D,i}$	Penetrant concentrations in the Henry’s region
$C_{H,i}$	Penetrant concentration in the Langmuir’s region
$D_{D,i}$	Diffusivity in the Henry region
$D_{H,i}$	Diffusivity in the Langmuir region
$F_{i}$	Ratio between diffusivity in Henry’s region and Langmuir’s regions
$K_{i}$	Ratio between Dual-Sorption Mode Model parameters
$p_{u,i}$	Upstream partial pressure of component i
$p_{d,i}$	Downstream partial pressure of component i
***Standard Transport Model for Permeability***	
$L_{i}$	Mobility coefficient of component i
$L_{i,0}$	Infinite dilution mobility coefficient of component i
β	Plasticization factor
$M_{i}$	Penetrant molecular weight
$Z_{i}$	Penetrant compressibility factor
$\rho_{pol}$	Polymer density
ϱ	Size selectivity of the polymer
τ	Polymer-dependent parameter in the empirical correlation between penetrant mobility and critical volume
$\varrho_{0}$	Pre-exponential factor for parameter η
$V_{c}$	Critical volume of penetrant
***Widom insertion method***	
$\mu_{i}^{ex}$	Excess chemical potential of penetrant i inside the polymer
$\Delta U_{\mathrm{test}}^{\mathrm{inter}}$	Potential energy of interaction between the test molecule and the other molecules
***Simulation of diffusivity***	
$\left\langle (r_{i}\left(t\right)-r_{i}\left(0\right){)}^{2}\right\rangle$	Mean squared displacement (MSD) averaged over all molecules
*t*	Time
$D_{i,self}^{MD}$	Self-diffusion coefficient computed in the MD simulations
η	Shear viscosity computed in MD simulations
Ξ	Dimensionless constant equal to 2.837298 for periodic (cubic) lattices
Λ	Simulation box length

## Appendix C. Self-Diffusion Coefficient (Mobility), Mutual-Diffusion Coefficient, and Thermodynamic Factor

The diffusive flux $J_{i}$, which is the actual driving force for diffusion, can be expressed either in terms of concentration gradient $\nabla \omega_{i}$ or penetrant chemical potential gradient $\nabla \mu_{i}$:

(A1) $$J_{i}=-D_{i}\rho \nabla \omega_{i}=-L_{i}\frac{\omega_{i}\rho }{RT}\nabla \mu_{i}$$

In the first expression, the mutual diffusion coefficient $D_{i}$ appears, while in the second one, the mobility, $L_{i}$, or self-diffusion coefficient, is used. Mobility has a purely kinetic meaning and is related to the resistance of molecular motion in the solid mixture [92,93,94,95]. As a consequence of this equivalence, the mutual diffusion coefficient $D_{i}$ can be rewritten as:

(A2) $$D_{i}=\frac{L_{i}}{RT}\frac{\partial \mu_{i}}{\partial \ln\omega_{i}}$$

The chemical potential of i can be related to the respective reference state value, $\mu_{i,ref}$, and ultimately to the activity $a_{i}$ as follows:

(A3) $$\mu_{i}=\mu_{i,ref}+RT\ln(f_{i}/f_{i,ref})=\mu_{i,ref}+RT\ln\left(a_{i}\right)$$

Thus, the relation between the mutual diffusion coefficient and mobility can be rewritten as:

(A4) $$D_{i}=L_{i}\frac{\partial \ln\alpha_{i}^{T}}{\partial \ln\omega_{i}}$$

where the term multiplying the mobility is defined as $\alpha^{T}$, the *thermodynamic factor*, as it is entirely thermodynamic in nature:

(A5) $$\alpha_{i}^{T}\equiv \frac{\partial \ln\mu_{i}}{\partial \ln\omega_{i}}$$

Based on the above equations, mutual diffusivity can be decomposed into this purely thermodynamic factor and the purely kinetic mobility term as follows:

(A6) $$D_{i}=L_{i}\alpha_{i}^{T}$$

Calculating the thermodynamic factor is possible either by using a thermodynamic model able to predict the gas solubility isotherm in the polymer membrane, or by taking experimental solubility isotherms for the specific fluid–polymer system. In the above equations, the activity coefficient $a_{i}$ can be replaced by the component fugacity $f_{i}$ if it is a real gas, or by its partial pressure $p_{i}$ if it is an ideal gas.
